# Supplementary material for: Effective remediation programs for vulnerable students to overcome learning loss
Source: PLoS One. 2025 May 14;20(5):e0323352. doi: 10.1371/journal.pone.0323352 (PMC12077795; doi:10.1371/journal.pone.0323352)
Supplement: S1 Appendix — (PDF) [file pone.0323352.s001.pdf]

S1 Appendix. Data collection.

1. Data collection

The Netherlands Cohort Study on Education (NCO) stored at Statistics Netherlands (CBS) includes information on all students in primary and secondary education in the Netherlands and background information about the student and the school. For this paper, we use data from several sources, which will be discussed in detail below:

1. NCO data of primary education in 2019/2020 and 2020/2021.
2. NCO data of student background information for 2019/2020 and 2020/2021.
3. NCO data of school-level background information for 2019/2020 and 2020/2021.
4. Standardized test data retrieved from the pupil monitoring systems for 2019/2020 and 2020/2021.
5. Data on remediation programs,
    a. Participation lists of students partaking in the remediation programs
    b. Information about the characteristics of remediation program retrieved from the fund application.

1.1 Primary education data

The NCO data consists of cohorts of students in primary education from 2010/2011 onwards. These data capture the trajectory of the students throughout primary education with information on the grade and the school in which the students follow education. This paper uses information about the students enrolled in primary education (kindergarten up until grade 6) in 2019/2020 and 2020/2021.

1.2 Student background data

Background data on the students is retrieved from the NCO register data, including information on the student (sex and migration background) and the students' (legal) parents. The parental information consists of income and educational attainment. Data from 2019/2020 and 2020/2021 are matched to the education cohorts.

1.3 School information data

Information related to the school characteristics can also be found in the NCO register data. It consists of information on the school identifiers, the urbanization level of the area, the denomination, and the percentage of disadvantaged students. Data from 2019/2020 and 2020/2021 are matched to the schools where the students conducted the tests.

1.4 Standardized test data

The standardized test data consists of test score data on reading and mathematics. In the Netherlands, students from grade one (at the age of six) start taking standardized tests twice a year. Once halfway into the school year, called the midyear test and the end-of-year test at the end of the school year. Dutch schools are required to take these tests and administrate these standardized tests into student monitoring systems. Primary schools are required to register the test scores into these student monitoring systems. The standardized test data are gathered from these student monitoring systems. For the data collection, we collaborated with three monitoring systems, CITO-LOVS, ParnasSys, and ESIS, and exported the test scores of students who took the CITO standardized test. CITO is the largest supplier in the Netherlands regarding primary education tests

in grades one to six. With the permission of schools and the possibility of parents objecting to export, the monitoring systems export the data to Statistics Netherlands. Statistics Netherlands then pseudonymizes the student-id and school-id. The test admin data can be combined with the Netherlands Cohort Study on Education with the student-id and school-id.

We have information on these test scores for reading and mathematics. The comprehensive reading test assesses the student's ability to understand written texts, including factual and literary content. The mathematics test contains abstract and contextual problems that describe a concrete task.

To compare the test scores across domains and compute an average composite score, the test scores were standardized by grade level (grade one to grade five; kindergarten and grade six are excluded). The test scores are standardized on the entire population of students, of which test scores are available to the researchers. We use the end-of-year test scores made at the end of the school year 2019/2020 (test score t_0) and the end-of-year test at the end of the school year 2020/2021 (test score t_1). In grade six, students take a different, standardized exit test that determines the track in secondary education, which is why this test is excluded as it is not comparable to the other tests taken throughout primary education. In kindergarten, students do not take these tests yet; they start taking tests in grade 1.

1.5 Remediation program data

1.5.1 Participation lists

When applying for the funds to set up remediation programs, schools were asked to participate in this study and deliver a list of students who participated in the remediation program. The schools provided the researchers with a list of (anonymized) students, with information on the student's

sex, date of birth, postal code, and house number. This information is essential to match the students to the register data of Statistics Netherlands (and thus to match them to the NCO register data and the test scores). A dataset with all the combined participation lists were then uploaded to Statistics Netherlands and matched to their unique identifier, with a success rate of 92%.

1.5.2 Characteristics of remediation program

Additionally, we have information about the characteristics of the remediation program. Schools were required to complete a questionnaire detailing their proposed remediation program to secure the funds for the remediation programs. There are several questions in this questionnaire we used for the analyses.

- Question 1: The first question focused on the program's content and included a drop-down menu with predefined options, allowing schools to select as many as were applicable. The question asked, "What type of program are you planning to offer?" with options including the purchase of additional teaching materials for monitoring, additional support and guidance, sports/games/culture, behaviour and communication, parental involvement, expanding digital education, creating successful experiences, developing study strategies, and summer or holiday school. If a school selected “Other, namely,” a text box immediately appeared for them to specify the type of support they intended to provide.
- Question 2: Additionally, if a school selected “Additional support and guidance,” a follow-up question appeared with a second drop-down menu, offering further details about the specific support measures the school could offer. These options included extended school days, extended or additional instruction, remedial teaching in small groups, guided practice

and automatization, pre-teaching, additional individual support, homework guidance, and extra support during independent work.

- Question 3: Schools were asked to provide insight into when the remediation efforts would take place. They could indicate whether the program occurred during regular school hours, outside of regular school hours, or during and outside of regular school hours.
- Question 4: Schools needed to indicate the size of the remediation program and the size of the groups receiving additional support. This could vary from entire classrooms to individual guidance. Schools could choose from the following options: whole class/grade, small groups of two to five students, small groups of six to ten students, groups of unknown size, individual support, individual support as well as in small groups, or unknown.
- Question 5: The remediation programs were established to enhance skills or achieve specific objectives. The following goals were outlined: mathematics goal, language goal, general school performance goal, social-emotional goal, study skills goal, teacher professionalization goal, sports goal, and a cultural/excursions/creative goal.
- Question 6: Supervisors guide and assist students throughout the remediation program. Different supervisors are available depending on whether schools collaborate with other institutions or if the program is outsourced. Supervisors may be internal, external, or a mix of both. Internal supervisors may consist of teachers, educational support staff, or teaching assistants; however, this distinction will not be made in the analyses as it was filled in quite poorly.
- Question 7: Schools were also requested to list potential collaborations with other institutions. These external collaborations could include partnerships with other schools, businesses, cultural institutions, municipalities, educational consultancy firms, sports

associations, or teacher training colleges. Schools should indicate whether they solely work with their internal professionals, fully rely on external professionals from these other organizations, or use a combination of both.

Based on this questionnaire, we received information about the characteristics of the remediation programs. We use the following information regarding the remediation programs: the organization, the timing, the group size, the goals, and the type of support offered by the programs. As for organization, schools can choose to organize it by themselves or collaborate with external parties, leading to a difference in whether internal or external staff are deployed. The program can take place during or outside regular school hours (regular hours in primary education are typically between 8:30 am and 3 pm). Furthermore, support is offered to students of different group sizes. The program goals are math, reading (or both), or non-cognitive skills such as study and socio-emotional skills. Lastly, the type of support includes additional support and guidance, purchase of new methods, extended instructions, extended school days, remedial teaching, support during work, or unknown/else.

1.6 Combining all data

The starting point is data on student's test scores. To all students of which we have test scores throughout primary education, we match the information from the participation lists. This implies that for some students and schools, we are unsure whether they had a remediation program or not, as we do not know the students who participated. These schools could have offered a program but did not send us the data. Or it could be that they did not have a program. Therefore, these students and schools are not used in our main analyses. The next step is to match the information regarding

the characteristics of the remediation programs. This is an in-depth analysis of the mechanisms of the programs. This means that in order to conduct these analyses, we need the student test scores, and schools need to send us their participation lists, as well as information on the characteristics of the programs offered. We cannot use schools in our analyses (and, therefore, their students) if we do not have a list of participating students.
