# Supplementary material for: Effective remediation programs for vulnerable students to overcome learning loss
Source: PLoS One. 2025 May 14;20(5):e0323352. doi: 10.1371/journal.pone.0323352 (PMC12077795; doi:10.1371/journal.pone.0323352)
Supplement: S2 Appendix — (PDF) [file pone.0323352.s002.pdf]

S2 Appendix. Representativeness of the sample.

More information about the sample's representativeness compared to the entire student population (retrieved from the Netherlands Cohort Study on Education [NCO]) in primary education in the Netherlands can be found in **Table 1**. Since the sample size is quite large, slight differences can already be statistically significant; therefore, we standardized all variables so that differences above 0.05 SD show the most important differences between the sample and the national average. Our sample over-represents vulnerable and disadvantaged students compared to the entire student population in primary education in the Netherlands. Our sample consists of more non-western migrant students, students with low-educated parents, low-income families, and one-parent families compared to the national averages. Furthermore, schools in our sample are located in more urbanized areas and have a somewhat higher disadvantage score. However, this overrepresentation is understandable, considering that remediation programs target vulnerable and disadvantaged students. In one of our robustness analyses, we calculated the Inverse Probability Weights (IPW) to correct the overrepresentation of vulnerable and underprivileged students.

Table 1. Representativeness of the sample compared to the entire student population in primary education in the Netherlands.

	Sample		NCO				
	**Mean**	**N**	**Mean**	**N**	**Diff.**	**T**	**P-value**
Girls	0.012	66,459	-0.000	1,954,682	0.013	3.177	0.001
Household structure							
Two-parent family	-0.018	66,459	0.001	1,954,682	-0.019	-4.756	0.000
One-parent family	0.037	66,459	-0.001	1,954,682	0.038	9.743	0.000
Migration background							
Dutch background	-0.077	66,459	0.003	1,954,682	-0.079	-20.146	0.000
Western background	0.087	66,459	-0.003	1,954,682	0.089	22.692	0.000
Non-western background	0.011	66,459	-0.000	1,954,682	0.012	2.935	0.003
Parental education level							
Low educated	-0.077	66,459	0.003	1,954,682	-0.079	-20.146	0.000
Average educated	0.087	66,459	-0.003	1,954,682	0.089	22.692	0.000
High educated	0.011	66,459	-0.000	1,954,682	0.012	2.935	0.003
Unknown	-0.073	66,459	0.002	1,954,682	-0.051	-13.013	0.000
Parental income level							
Low income	0.051	66,459	-0.002	1,954,682	0.053	13.322	0.000
Average income	0.009	66,459	-0.000	1,954,682	0.009	2.301	0.021
High income	-0.028	66,459	0.001	1,954,682	-0.029	-7.293	0.000
Parental labor market position							
Both parents work	-0.008	66,459	0.000	1,954,682	-0.009	-2.204	0.000
Only father works	0.003	66,459	-0.000	1,954,682	0.003	0.869	0.385
Only mother works	0.030	66,459	-0.001	1,954,682	0.031	7.826	0.000
Both parents don't work	0.031	66,459	-0.001	1,954,682	0.032	8.143	0.000
Denomination							
Public	0.161	66,459	-0.005	1,954,682	0.166	42.180	0.000
Based on educational philosophies	-0.120	66,459	0.004	1,954,682	-0.124	-31.503	0.000
Based on religious beliefs	-0.094	66,459	0.003	1,954,682	-0.097	-24.589	0.000
Urbanization of the school							
No urbanization	-0.135	66,459	0.005	1,954,682	-0.140	-35.532	0.000
Low urbanization	-0.171	66,459	0.006	1,954,682	-0.177	-44.951	0.000

Average urbanization	0.015	66,459	-0.001	1,954,682	0.015	3.896	0.000
Strong urbanization	0.134	66,459	-0.005	1,954,682	0.139	35.186	0.000
Very strong urbanization	0.092	66,459	-0.003	1,954,682	0.095	24.030	0.000
School disadvantage score	10.275	66,459	9.864	1,954,682	0.411	26.173	0.000
