# Supplementary material for: Effective remediation programs for vulnerable students to overcome learning loss
Source: PLoS One. 2025 May 14;20(5):e0323352. doi: 10.1371/journal.pone.0323352 (PMC12077795; doi:10.1371/journal.pone.0323352)
Supplement: S3 Appendix — (PDF) [file pone.0323352.s003.pdf]

S3 Appendix. Descriptive statistics and operationalization

3.1 Participating students

Students can be flagged as participating students using participation lists received from schools. All students enlisted in a school with participating students who are not on the participation list are labelled as 'non-participants'. All schools that did not provide participation lists are excluded from the sample. These schools did not offer remediation programs for the students or did not share the participation list with the researchers.

In total, we have information about 456 schools in the Netherlands, a total sample of 66,439 students, of which 10,704 students have participated in remediation programs, and 55,735 students have not participated in the remediation programs.

3.2 Test scores

The scores on the standardized tests during school years 2019/2020 and 2020/2021 are used for this paper. **Figure 1** graphically shows the testing moments and the school closures over these two years. Before using the test scores, several data-cleaning steps are taken. We only include students who are officially registered in the Netherlands. Test scores must have valid scores within the acceptable range. Duplicate observations were removed if they were identical but taken at different schools. Only test scores from supplier CITO are considered valid. After the cleaning, the test scores are standardized. The scores are standardized based on domain and grade level in primary education as the growth is not linear over all grade levels. Students in specific grades can achieve more growth than students in other grades.

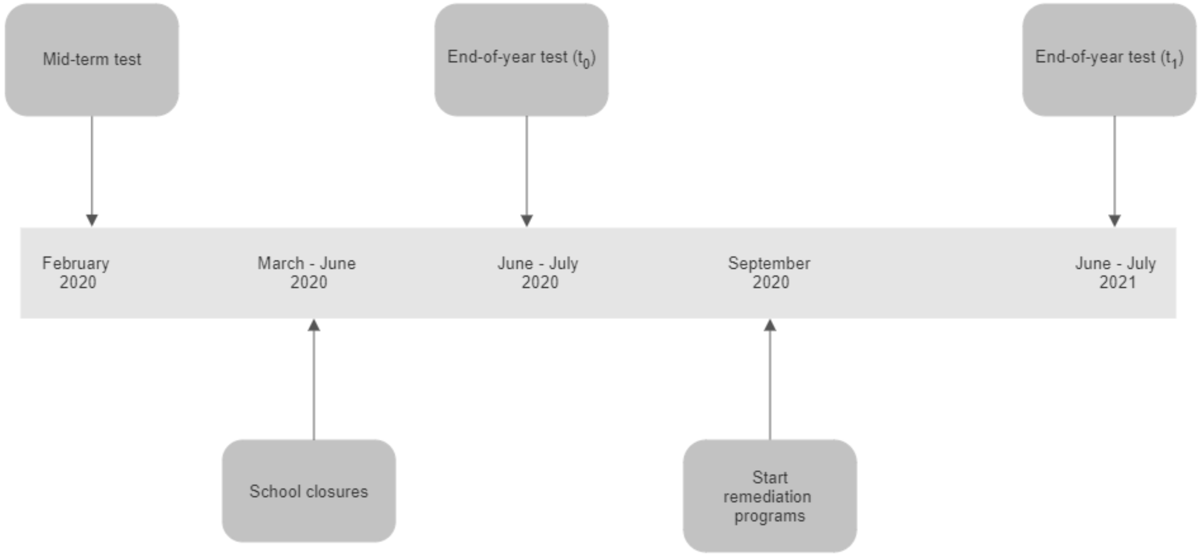

Figure 1: Timing of tests and school closures in the Netherlands during 2019/2020 and 2020/2021.

Furthermore, we noticed that pupils are tested less or that testing is postponed after the first school closures. The first school closure had consequences for the end-of-year test of 2020 (t0), as many schools delayed testing their students. Some schools decided to test the students in July or the beginning of August instead of June. Other schools chose to test the students after the summer holidays (August, September, or even October); around 30% of the reading and 22% of the mathematics tests were taken after the summer holidays. The descriptive statistics of the standardized test scores are presented in **Table 1.**

Table 1. Descriptive statistics about students' test scores separated by participating and non-participating students in remediation programs.

	N	Mean	Std. Dev.	Min. value	Max. value
Participating students					
End-of-year test t_0					
Reading	4,360	-0.482	0.921	-3.881	3.933
Mathematics	4,360	-0.491	0.984	-5.134	3.374
Composite score	4,360	-0.486	0.826	-3.690	3.359
End-of-year test t_1					
Reading	6,344	-0.417	0.940	-4.124	5.642
Mathematics	6,344	-0.425	0.998	-5.272	4.432
Composite score	6,344	-0.421	0.851	-4.001	3.522
Non-participating students					
End-of-year test t_0					
Reading	22,861	0.049	0.989	-3.970	5.640
Mathematics	22,861	0.068	0.973	-7.314	4.432
Composite score	22,861	0.059	0.865	-4.268	3.908
End-of-year test t_1					
Reading	32,874	0.064	0.991	-4.610	5.642
Mathematics	32,874	0.075	0.972	-8.284	4.793
Composite score	32,874	0.069	0.874	-5.609	4.186

3.3 Individual-level background variables

The data distinguish between pupils' background variables such as sex, migration background, parental education level, parental income level, parental employment status, socio-economic status, and family structure. Descriptive statistics can be found in **Table 2.**

- *Grade*: The student will be in primary school in 2019/2020 in grades 1, 2, 3, 4, 5, or 6.
- *Sex*: girl (1) or boy (0).
- *Migration background*: whether the student has a Dutch background (0), a Western background (1), or a non-Western background (2). Students with non-Western backgrounds have a background from all countries in Africa, Latin America, Asia (excluding Indonesia and Japan), or Turkey.

- *Parental education attainment*: parental education is defined as low when the highest obtained degree of (one) the parents is in pre-vocational secondary education (vmbo b/k), or a degree in upper secondary vocational education (mbo 1), or grades 7 to 9 in pre-vocational secondary education (vmbo gl/tl) or senior general secondary education or university preparatory education (1), middle when a degree in upper secondary vocational education level 2, 3 or 4, or when completed senior general secondary education or university preparatory education (2), and high when a degree at a university of applied sciences is attained or higher (3).
- *Parental income level*: Based on parental income, three categories are made: low is when the parental income is below the modal income (0), average is when the parental income is between modal income and twice modal income (1), and high is when the parental income is above two times the modal income (2).
- *Household type*: a distinction between students in two-parent families (0) and one-parent families. A one-parent family is when the students are officially registered with only one legal parent. Students who do not live with either of their parents, for example, live in a youth house, are excluded from the sample (about 0.7% of the population).
- *Labor market position of parents*: the employment status consists of four categories: either both parents work (0), only the father is active in the labor market (1), only the mother is active in the labor market (2), or neither of the parents is active on the labor market (3). Not being active in the labor market means the parents do not receive income from paid work.

Table 2. Descriptive statistics showed separately for participating and non-participating students in remediation programs and the remainder of the Dutch primary school population.

	**Non-participants**		**Participants**	
	**N**	**%**	**N**	**%**
Gender				
Girls	27,264	48.92	5,481	51.21
Boys	28,471	51,08	5,223	48.79
Migration background				
Dutch background	39,770	71.36	6,683	62.43
Western background	11,202	20.10	3,069	28.67
Non-western background	4,763	8.55	952	8.89
Parental education level				
Low educated	4,90	8.78	1,610	15.04
Average educated	16,893	30.11	3,894	36.38
High educated	29,058	52.14	4,033	37.68
Unknown	4,884	8.76	1,167	10.90
Parental income level				
Low income	11,479	20.60	3,221	30.09
Average income	29,866	53.59	5,551	51.86
High income	14,160	25.41	1,873	17.50
Household structure				
Two-parent family	46,138	82.78	8,420	78.66
One-parent family	9,597	17.22	2,284	21.34
Parental labor market position				
Both parents work	41,067	77.91	6,762	68.75
Only father works	6,897	13.09	1,647	16.75
Only mother works	2,219	4.21	521	5.30
Both parents don't work	2,374	4.50	864	8.78
Grade in primary education				
Grade 2	14,888	26.71	2,988	27.91
Grade 3	16,214	29.09	3,048	28.48
Grade 4	16,346	29.33	3,083	28.80
Grade 5	8,287	14.87	1,585	14.81

3.4 School-level background variables

School-level background variables include pupil-population variables and school characteristics. **Table 3** provides descriptive statistics about these variables**.**

- *Denomination*: A school's denomination can be a public one (0), a religious/philosophical affiliation (1) (most common – e.g., Catholic, Protestant, Muslim), or an educational or pedagogical concept (2) (e.g., Montessori, Dalton), or a combination of both, such as Waldorf schools (3). While a combination of denominations rarely occurs in our data, it is therefore not included in our sample.
- *School-disadvantage-score*: The score expresses a student's risk of educational disadvantage: the higher the score, the greater the risk of educational disadvantage. The score represents a range from 1-100 in percentile scores. This score is then calculated at the school level and expresses the expected educational disadvantage in schools.
- *Urbanization level of the school*: the place in which the school is located has a specific urbanization level; we classify five different levels where either the school is placed in an area with no urbanization (0), low urbanization (1), some urbanization (2), strong urbanized areas (3) and very urbanized areas (4).

Table 3. Descriptive statistics of school-level variables.

	**Participants**		**Non-participants**	
	**N**	**%**	**N**	**%**
Denomination				
Public	4,098	38.28	20,724	37.18
Based on educational philosophies	137	1.28	1,747	3.13
Based on religious beliefs	6,469	60.44	33,264	59.68
Urbanization of the school				
No urbanization	363	3.39	2,265	.06
Low urbanization	1,578	14.74	8,117	14.56
Average urbanization	1,627	15.20	9,599	17.22
Strong urbanization	4,777	44.63	19,939	35.77
Very strong urbanization	2,359	22.04	15,815	28.38
	**Participants**		**Non-participants**	
	**Mean**	**SD**	**Mean**	**SD**
School-disadvantage score	54.054	29.996	48.406	29.716

3.5 Characteristics of remediation program

Schools had to complete a questionnaire detailing their proposed remediation program to secure the funds for the remediation programs. Based on this questionnaire, we received information about the characteristics of the remediation programs. More information and descriptive statistics can be found in **Table 4**.

Note that we only know the characteristics of a subset of students/schools. Our sample consists of N = 66,439 students, of which N = 55,735 are non-participating students in remediation programs. The remaining N = 10,704 students participate in remediation programs, and for some, we have information about the characteristics of this remediation program. We have information about the characteristics of the remediation programs for N = 3,947 participating students in remediation programs. For the other N = 6,757 students, we know these students did participate in the program, but we have not received information about the content of the programs. These students will be included in the analyses as a separate category. Table 4 shows the percentages of the different characteristics for the *participating* students (N = 10,704).

We distinguish between the following aspects of the program:

- *Moment of the program*: Schools can set up the remediation program during different times, namely (1) remediation program offered during school, (2) remediation program offered after school, (3) remediation program offered during and after school, (4) unknown, where the school has not answered this question. Note that the regular school day hours in the Netherlands are from 8:30/9:00 to 15:00/15:30.
- *Organization of the program*: Both internal and external parties can help organize the remediation program. This variable captures the organization of the remediation program based on three aspects: the supervisors, executors, and outsourcers. These three variables

are combined to create a new variable, the new organization variable consists of (1) only internal staff (such as teachers, assistants, interns), (2) only external staff, (3) a combination of internal and external staff, or (4) unknown, when the school has not provided any detail on the organization of their remediation program.

- *Goal of the program*: The goals the remediation program wanted to reach can be clustered into (1) cognitive goals (math and language goals), (2) language goals, (3) mathematic goals (4) cognitive and non-cognitive goals, or (5) non-cognitive goals (socio-emotional or study skills).
- *Group size:* The group size variable consists of the number of students per group for whom the remediation program is offered simultaneously. The variable distinguishes between (1) for the entire class, (2) for groups with 2 to 5 students, (3) for groups with 6 to 10 students, (4) groups with an unknown number of students, (5) individual programs, (6) individual and group support, and (7) unknown, where the school has not answered this question.
- *Type of support offered during the program*: this is a variable based on two questions in the questionnaire: first, the "what" and second, the "if extra support and guidance, then:". This variable, type of support, is constructed based on answers of the two questions. To create a variable that categorizes the types of support offered, we looked at what types of support are offered, how many schools chose for certain types of support and whether they are offered in combination with other types of support or not. For the first question of the questionnaire, concerning "what" was offered, schools mostly offer "additional teaching materials" or "additional support and guidance". For the second question, the follow up question regarding which extra support and guidance is offered, schools often chose "extended instructions", "extended schooldays", "additional support during independent

work”, or “remedial teaching”, or combinations of these. Additionally, many schools answered “other” or left the entire second question open. Based upon these insights, we have created the following categorization: (1) additional support and guidance [without a further specification of what this additional support entails], (2) purchase of new methods [these new methods are mainly monitoring tools for teachers], (3) extended instructions [including pre-teaching and guided automation and practice], (4) extended school day [including homework supervision], (5) remedial teaching [including individual help], (6) support during individual work, (7) other [often interventions that only a small fraction of the schools chose, for example, summer schools or sport and cultural programs], or (8) unknown/not filled in.

Table 4. Descriptive statistics about the elements of remediation programs for participating students.

	% of participating students
Moment of remediation program	
Program outside regular hours	4.10%
Program during regular hours	4.83%
Program outside & during	10.35%
Unknown/not filled in	17.68%
Students without information	63.03%
Organization of remediation program	
Internal staff	15.64%
External staff	2.10%
Internal & external staff	18.41%
Unknown/not filled in	0.81%
Students without information	63.03%
Group size	
Entire class	1.37%
Small groups (2-5 students)	2.43%
Small groups (6-10 students)	0.64%
Groups of unknown size	7.99%
Individual	0.77%
Individual and groups	4.24%
Unknown/not filled in	19.52%
Students without information	63.03%
Type of support offered	
Additional support and guidance	11.01%
Purchase of new methods	5.35%
Extended instructions	3.92%
Extended school day	2.73%
Remedial teaching	0.79%
Support during work	0.76%
Unknown/not filled in	12.37%
Students without information	63.03%
Goal	
Language and math goals	15.73%
Language goals	3.20%
Mathematic goals	1.60%
Cognitive and non-cognitive	14.87%
Non-cognitive goals	1.58%
Students without information	63.03%
