# Supplementary material for: Effective remediation programs for vulnerable students to overcome learning loss
Source: PLoS One. 2025 May 14;20(5):e0323352. doi: 10.1371/journal.pone.0323352 (PMC12077795; doi:10.1371/journal.pone.0323352)
Supplement: S4 Appendix — (PDF) [file pone.0323352.s004.pdf]

S4 Appendix. PSM-matching approach.

We use a PSM-matching model for the robustness check. Our propensity score matching approach incorporates several student background characteristics to match participating and non-participating students. We use the students' test scores from January/February/March of the school year 2019/2020, right before the school closures due to COVID-19. Furthermore, we included several background characteristics of the students: gender, migration background, household structure, education level of the parents, the income of the parents, and lastly, the parents' working status.

We estimated several different PSM models with all these background characteristics: Kernel logit matching with different bandwidths (0.06 default; 0.02 and 0.12). The Kernel matching allows us to weigh observations depending on their proximity to the treatment group based on all the background characteristics, with the main advantage that we hardly lose any students in our sample. The different bandwidth options allow for a higher or lower inclusion rate. This means that a larger bandwidth allows a greater distance between the matches, which leads to a larger inclusion of cases in the matching, although it also increases the risk of bias. The default setting in Stata is 0.06; we have included a somewhat more restricted value and a more lenient value. With this approach, we can use almost all students in our model, and these students receive a weight that can be added to our Difference-in-Differences model.

Tables 1, 2, and 3 show the results of the analyses with PSM-weights (kernel, with different bandwidths) for the composite score, reading score and mathematics score, including both the student and school-level control variables. Additionally, **Table 4** shows the analyses with half of the sample, including the PSM-weights as an additional robustness check.

Table 1. Student achievements before and after remediation programs with PSM-matching weights (Kernel bandwidth 0.02).

	Composite score	Reading score	Mathematics score
School year 2020/2021[a]	0.019^	0.028*	0.007
	(0.011)	(0.011)	(0.012)
Participating students[b]	-0.559***	-0.529***	-0.566***
	(0.025)	(0.026)	(0.028)
School year * Participation	0.047**	0.054*	0.042*
	(0.018)	(0.021)	(0.019)
Student controls	Yes	Yes	Yes
School level controls	Yes	Yes	Yes
Clustered standard errors	Yes	Yes	Yes
School dummies	Yes	Yes	Yes
Constant	-0.419*	-0.567**	-0.131
	(0.185)	(0.200)	(0.180)
Observations	48,223	48,858	60,809
R-squared	0.271	0.244	0.239

Note: Robust standard errors in parentheses; *** $p < 0.001$, ** $p < 0.01$, * $p < 0.05$, ^ $p < 0.1$. [a] the reference category is the school year 2019/2020; [b] the reference category is students who did not participate in the remediation programs but are enrolled in schools that offer remediation programs. Student controls include sex, migration background, parental education and income, and household structure; school-level controls include denomination, urbanization, and the disadvantage score of the school.

Table 2. Student achievements before and after remediation programs with PSM-matching weights (Kernel bandwidth 0.06).

	Composite score	Reading score	Mathematics score
School year 2020/2021[a]	0.018^	0.025*	0.006
	(0.011)	(0.011)	(0.012)
Participating students[b]	-0.562***	-0.533***	-0.567***
	(0.025)	(0.026)	(0.027)
School year * Participation	0.049**	0.056**	0.043*
	(0.018)	(0.021)	(0.019)
Student controls	Yes	Yes	Yes
School level controls	Yes	Yes	Yes
Clustered standard errors	Yes	Yes	Yes
School dummies	Yes	Yes	Yes
Constant	-0.413*	-0.553**	-0.131
	(0.185)	(0.200)	(0.180)
Observations	48,223	48,858	60,809
R-squared	0.275	0.247	0.242

Note: Robust standard errors in parentheses; *** $p < 0.001$, ** $p < 0.01$, * $p < 0.05$, ^ $p < 0.1$. [a] the reference category is the school year 2019/2020; [b] the reference category is students who did not participate in the remediation programs but are enrolled in schools that offer remediation programs. Student controls include sex, migration background, parental education and income, and household structure; school-level controls include denomination, urbanization, and the disadvantage score of the school.

Table 3. Student achievements before and after remediation programs with PSM-matching weights (Kernel bandwidth 0.12).

	Composite score	Reading score	Mathematics score
School year 2020/2021[a]	0.015	0.023*	0.003
	(0.010)	(0.011)	(0.011)
Participating students[b]	-0.569***	-0.542***	-0.574***
	(0.025)	(0.026)	(0.027)
School year * Participation	0.052**	0.059**	0.046*
	(0.018)	(0.021)	(0.019)
Student controls	Yes	Yes	Yes
School level controls	Yes	Yes	Yes
Clustered standard errors	Yes	Yes	Yes
School dummies	Yes	Yes	Yes
Constant	-0.405*	-0.539**	-0.123
	(0.185)	(0.201)	(0.179)
Observations	48,223	48,858	60,809
R-squared	0.284	0.253	0.248

Note: Robust standard errors in parentheses; *** $p < 0.001$, ** $p < 0.01$, * $p < 0.05$, ^ $p < 0.1$. [a] the reference category is the school year 2019/2020; [b] the reference category is students who did not participate in the remediation programs but are enrolled in schools that offer remediation programs. Student control variables include sex, migration background, parental education and income, and household structure; school-level control variables include denomination, urbanization, and the disadvantage score of the school.

Table 4. Student's achievements before and after remediation programs with lower performing students only (half of the sample with PSM-weights; Kernel bandwidth = 0.06)

	Composite score	Reading score	Mathematics score
School year 2020/2021[a]	0.029**	0.057***	0.062***
	(0.010)	(0.011)	(0.011)
Participating students[b]	-0.294***	-0.260***	-0.311***
	(0.015)	(0.017)	(0.017)
School year * Participation	0.052**	0.043*	0.013
	(0.019)	(0.021)	(0.021)
Student controls	Yes	Yes	Yes
School level controls	Yes	Yes	Yes
Clustered standard errors	Yes	Yes	Yes
School dummies	Yes	Yes	Yes
Constant	-0.628***	-0.741***	-0.392*
	(0.150)	(0.170)	(0.160)
Observations	25,192	25,767	31,210
R-squared	0.178	0.154	0.163

Note: Robust standard errors in parentheses; *** $p < 0.001$, ** $p < 0.01$, * $p < 0.05$, ^ $p < 0.1$. [a] the reference category is the school year 2019/2020; [b] the reference category is students who did not participate in the remediation programs but are enrolled in schools that offer remediation programs. Student control variables include sex, migration background, parental education and income, and household structure; school-level control variables include denomination, urbanization, and the disadvantage score of the school.
