# Supplementary material for: Effective remediation programs for vulnerable students to overcome learning loss
Source: PLoS One. 2025 May 14;20(5):e0323352. doi: 10.1371/journal.pone.0323352 (PMC12077795; doi:10.1371/journal.pone.0323352)
Supplement: S1 Table — (PDF) [file pone.0323352.s005.pdf]

S1 Table. The likelihood of participating in remediation programs (learning loss indicator).

	M1 Comp	M2 Reading	M3 Math
Grade[a]			
2nd grade	0.122	0.120	0.120
	(0.100)	(0.100)	(0.100)
4th grade	0.113	0.114	0.111
	(0.076)	(0.076)	(0.076)
Girls	0.106**	0.104**	0.109**
	(0.039)	(0.039)	(0.039)
Migration background[b]			
Western migrant	0.189^	0.184^	0.189^
	(0.102)	(0.102)	(0.102)
Non-western migrant	0.062	0.057	0.063
	(0.085)	(0.085)	(0.086)
Parental education level[c]			
Low educated	0.215**	0.220***	0.212**
	(0.066)	(0.066)	(0.066)
High educated	-0.368***	-0.371***	-0.373***
	(0.096)	(0.097)	(0.097)
Parental income level[d]			
Low income	0.108	0.110^	0.112^
	(0.067)	(0.067)	(0.067)
High income	-0.111	-0.114	-0.118
	(0.087)	(0.087)	(0.087)
Parental labor market position[e]			
Only father works	0.094	0.096	0.096
	(0.063)	(0.063)	(0.063)
Only mother works	0.152^	0.156^	0.153^
	(0.091)	(0.091)	(0.091)
Both parents don't work	0.228*	0.240*	0.228*
	(0.106)	(0.107)	(0.106)
Household structure[f]			
One-parent family	0.024	0.026	0.024
	(0.062)	(0.062)	(0.062)
Previous performance	-0.183***	-0.107**	-0.101**
	(0.055)	(0.035)	(0.035)
Constant	-1.749***	-1.742***	-1.743***
	(0.113)	(0.112)	(0.112)
Observations	20,895	20,895	20,895
Clusters	394	394	394

Standard errors in parentheses; *** p < 0.001, ** p < 0.01, * p < 0.05, ^ p < 0.1; [a] the reference category is the 3rd grade; [b] reference category is students with a Dutch background; [c] the reference category is an average parental education; [d] the reference category is average parental income; [e] reference category is students of which both parents work; [f] the reference category is a two-parent family. The measure of previous performance is based on learning loss between the midyear test and end-year test.
