# Supplementary material for: Effective remediation programs for vulnerable students to overcome learning loss
Source: PLoS One. 2025 May 14;20(5):e0323352. doi: 10.1371/journal.pone.0323352 (PMC12077795; doi:10.1371/journal.pone.0323352)
Supplement: S2 Table — (PDF) [file pone.0323352.s006.pdf]

S2 Table. The likelihood of participating in remediation programs (end-of-year test scores).

	M1 Comp	M2 Reading	M3 Math
Grade[a]			
1st grade	0.101	0.090	0.104
	(0.156)	(0.155)	(0.154)
2nd grade	0.051	0.045	0.057
	(0.094)	(0.093)	(0.094)
4th grade	0.079	0.101	0.073
	(0.077)	(0.076)	(0.077)
Girls	0.045	0.221***	-0.093**
	(0.035)	(0.040)	(0.034)
Migration background[b]			
Western migrant	0.061	0.081	0.106
	(0.007)	(0.096)	(0.097)
Non-western migrant	0.036	0.020	0.066
	(0.077)	(0.076)	(0.076)
Parental education level[c]			
Low educated	0.125^	0.130*	0.152*
	(0.065)	(0.065)	(0.064)
High educated	-0.106	-0.167*	-0.177*
	(0.075)	(0.077)	(0.078)
Parental income level[d]			
Low income	0.065	0.079	0.074
	(0.061)	(0.061)	(0.061)
High income	-0.025	-0.050	-0.052*
	(0.074)	(0.074)	(0.073)
Parental labor market position[e]			
Only father works	0.034	0.055	0.047
	(0.058)	(0.057)	(0.059)
Only mother works	0.110	0.134	0.104
	(0.083)	(0.081)	(0.082)
Both parents don't work	0.111	0.125	0.153
	(0.097)	(0.096)	(0.096)
Household structure[f]			
One-parent family	-0.083	-0.056	-0.084
	(0.060)	(0.059)	(0.060)
Previous performance	-0.705***	-0.545***	-0.532***
	(0.064)	(0.050)	(0.049)
Constant	-1.880***	-1.930***	-1.758***
	(0.125)	(0.125)	(0.118)
Observations	26,614	26,614	26,614
Clusters	404	404	404

Standard errors in parentheses; *** p < 0.001, ** p < 0.01, * p < 0.05, ^ p < 0.1; [a] the reference category is the 3rd grade; [b] reference category is students with a Dutch background; [c] the reference category is an average parental education; [d] the reference category is average parental income; [e] reference category is students of which both parents work; [f] the reference category is a two-parent family. The measure for previous performance is not learning loss, but the individual score on the end-of-the-year test in school year 2019/2020
