# Supplementary material for: Effective remediation programs for vulnerable students to overcome learning loss
Source: PLoS One. 2025 May 14;20(5):e0323352. doi: 10.1371/journal.pone.0323352 (PMC12077795; doi:10.1371/journal.pone.0323352)
Supplement: S3 Table — (PDF) [file pone.0323352.s007.pdf]

S3 Table. The likelihood of participating in remediation programs for the remediation programs sample.

	M1 Comp	M2 Reading	M3 Math
Grade[a]			
2nd grade	-0.067	-0.071	-0.066
	(0.158)	(0.159)	(0.159)
4th grade	0.095	0.099	0.092
	(0.113)	(0.114)	(0.113)
Girls	0.099	0.101	0.101
	(0.070)	(0.070)	(0.070)
Migration background[b]			
Western migrant	0.399**	0.397*	0.397**
	(0.154)	(0.155)	(0.154)
Non-western migrant	0.219	0.212	0.220
	(0.145)	(0.144)	(0.145)
Parental education level[c]			
Low educated	0.248*	0.254*	0.247*
	(0.107)	(0.107)	(0.107)
High educated	-0.477***	-0.482***	-0.481***
	(0.134)	(0.134)	(0.134)
Parental income level[d]			
Low income	-0.030	-0.022	-0.029
	(0.106)	(0.105)	(0.105)
High income	-0.187^	-0.191^	-0.190^
	(0.103)	(0.103)	(0.103)
Parental labor market position[e]			
Only father works	0.011	0.014	0.012
	(0.098)	(0.098)	(0.098)
Only mother works	0.053	0.052	0.052
	(0.165)	(0.165)	(0.164)
Both parents don't work	0.155	0.156	0.153
	(0.181)	(0.180)	(0.181)
Household structure[f]			
One-parent family	0.173	0.170	0.172
	(0.105)	(0.105)	(0.105)
Previous performance	-0.163^	-0.079	-0.106*
	(0.084)	(0.054)	(0.053)
Constant	-1.475***	-1.468***	-1.471***
	(0.194)	(0.193)	(0.192)
Observations	6,636	6,636	6,636
Clusters	135	135	135

Standard errors in parentheses; *** p < 0.001, ** p < 0.01, * p < 0.05, ^ p < 0.1 ; [a] the reference category is the 3rd grade; [b] reference category is students with a Dutch background; [c] the reference category is an average parental education; [d] the reference category is average parental income; [e] reference category is students of which both parents work; [f] the reference category is a two-parent family.
