# Supplementary material for: Effective remediation programs for vulnerable students to overcome learning loss
Source: PLoS One. 2025 May 14;20(5):e0323352. doi: 10.1371/journal.pone.0323352 (PMC12077795; doi:10.1371/journal.pone.0323352)
Supplement: S4 Table — (PDF) [file pone.0323352.s008.pdf]

S4 Table. The likelihood of participating in remediation programs for the cognitive remediation programs sample.

	M1 Comp	M2 Reading	M3 Math
Grade[a]			
2nd grade	-0.074	-0.078	-0.073
	(0.174)	(0.174)	(0.174)
4th grade	0.049	0.051	0.045
	(0.118)	(0.118)	(0.117)
Girls	0.101	0.101	0.103
	(0.077)	(0.077)	(0.077)
Migration background[b]			
Western migrant	0.309^	0.307^	0.307^
	(0.173)	(0.174)	(0.172)
Non-western migrant	0.084	0.080	0.085
	(0.160)	(0.159)	(0.160)
Parental education level[c]			
Low educated	0.311**	0.316**	0.312**
	(0.111)	(0.111)	(0.111)
High educated	-0.490**	-0.495***	-0.492**
	(0.150)	(0.150)	(0.150)
Parental income level[d]			
Low income	-0.086	-0.080	-0.084
	(0.117)	(0.116)	(0.117)
High income	-0.231*	-0.235*	-0.234*
	(0.111)	(0.111)	(0.111)
Parental labor market position[e]			
Only father works	0.048	0.049	0.050
	(0.109)	(0.109)	(0.109)
Only mother works	0.076	0.079	0.076
	(0.178)	(0.178)	(0.177)
Both parents don't work	0.117	0.121	0.113
	(0.203)	(0.203)	(0.202)
Household structure[f]			
One-parent family	0.204^	0.201^	0.203^
	(0.107)	(0.108)	(0.106)
Previous performance	-0.142	-0.079	-0.081
	(0.092)	(0.059)	(0.059)
Constant	-1.384***	1.374***	-1.380***
	(0.216)	(0.215)	(0.215)
Observations	5,572	5,572	5,572
Clusters	114	114	114

Standard errors in parentheses; *** p < 0.01, ** p < 0.05, * p < 0.1; [a] the reference category is the 3rd grade; [b] reference category is students with a Dutch background; [c] the reference category is an average parental education; [d] the reference category is average parental income; [e] reference category is students of which both parents work; [f] the reference category is a two-parent family.
