# Supplementary material for: Effective remediation programs for vulnerable students to overcome learning loss
Source: PLoS One. 2025 May 14;20(5):e0323352. doi: 10.1371/journal.pone.0323352 (PMC12077795; doi:10.1371/journal.pone.0323352)
Supplement: S6 Table — (PDF) [file pone.0323352.s010.pdf]

S6 Table. Student achievements before and after remediation programs – Reading score

	**Model 1**	**Model 2**	**Model 3**
School year 2020/2021[a]	0.011	0.015^	0.015^
	(0.008)	(0.008)	(0.008)
Participating students[b]	-0.606***	-0.514***	-0.514***
	(0.016)	(0.016)	(0.016)
School year * Participation	0.052*	0.050**	0.050**
	(0.020)	(0.019)	(0.019)
Student controls	No	Yes	Yes
School level controls	No	No	Yes
School-level fixed effects	Yes	Yes	Yes
Constant	0.063***	-0.444***	-0.447**
	(0.006)	(0.016)	(0.140)
Observations	66,439	66,439	66,439
R-squared	0.038	0.121	0.121
Clusters	456	456	456

Note: Robust standard errors in parentheses; *** p < 0.001, ** p < 0.01, * p < 0.05, ^ p < 0.1. [a] the reference category is the school year 2019/2020; [b] the reference category is students who did not participate in the remediation programs but are enrolled in schools that offer remediation programs. Student control variables include sex, migration background, parental education and income, and household structure; school-level control variables include denomination, urbanization, and the disadvantage score of the school.
