# Supplementary material for: Effective remediation programs for vulnerable students to overcome learning loss
Source: PLoS One. 2025 May 14;20(5):e0323352. doi: 10.1371/journal.pone.0323352 (PMC12077795; doi:10.1371/journal.pone.0323352)
Supplement: S8 Table — (PDF) [file pone.0323352.s012.pdf]

S8 Table. DiD-estimator separately, per grade, analyses student achievements before and after remediation programs.

	Composite	Reading	Mathematics
2nd grade to 3rd grade	0.038	0.043	0.037
	(0.029)	(0.033)	(0.033)
3rd grade to 4th grade	0.065**	0.075**	0.054^
	(0.023)	(0.026)	(0.029)
4th grade to 5th grade	0.065*	0.061*	0.070*
	(0.027)	(0.031)	(0.030)

Robust standard errors in parentheses; *** $p < 0.001$, ** $p < 0.01$, * $p < 0.05$, ^ $p < 0.1$. [a] the reference category is the school year 2019/2020; [b] the reference category is students who did not participate in the remediation programs but are enrolled in schools that offer remediation programs. Student control variables include sex, migration background, parental education and income, and household structure; school-level control variables include denomination, urbanization, and the disadvantage score of the school.
