# Supplementary material for: Effective remediation programs for vulnerable students to overcome learning loss
Source: PLoS One. 2025 May 14;20(5):e0323352. doi: 10.1371/journal.pone.0323352 (PMC12077795; doi:10.1371/journal.pone.0323352)
Supplement: S10 Table — (PDF) [file pone.0323352.s014.pdf]

S10 Table. Student achievements before and after remediation programs with lower-performing students only (half of the sample)

	Composite score	Reading score	Mathematics score
School year 2020/2021[a]	0.029**	0.057***	0.062***
	(0.010)	(0.011)	(0.011)
Participating students[b]	-0.294***	-0.260***	-0.311***
	(0.015)	(0.017)	(0.017)
School year * Participation	0.052**	0.043*	0.013
	(0.019)	(0.021)	(0.021)
Student controls	Yes	Yes	Yes
School level controls	Yes	Yes	Yes
School-level fixed effects	Yes	Yes	Yes
Constant	-0.701***	-0.840***	-0.478**
	(0.168)	(0.190)	(0.180)
Observations	25,237	25,767	31,245
R-squared	0.071	0.056	0.075
Clusters	435	436	445

Note: Robust standard errors in parentheses; *** $p < 0.001$, ** $p < 0.01$, * $p < 0.05$, ^ $p < 0.1$. [a] the reference category is the school year 2019/2020; [b] the reference category is students who did not participate in the remediation programs but are enrolled in schools that offer remediation programs. Student controls include sex, migration background, parental education and income, and household structure; school-level controls include denomination, urbanization, and the disadvantage score of the school.
