# Supplementary material for: Effective remediation programs for vulnerable students to overcome learning loss
Source: PLoS One. 2025 May 14;20(5):e0323352. doi: 10.1371/journal.pone.0323352 (PMC12077795; doi:10.1371/journal.pone.0323352)
Supplement: S11 Table — (PDF) [file pone.0323352.s015.pdf]

S11 Table. Effect of remediation program's group size on students' achievements.

	Composite	Reading	Mathematics
School year 2020/2021[a]	0.006	0.008	0.003
	(0.009)	(0.009)	(0.010)
Whole class[b]	-0.035	-0.021	-0.072
	(0.307)	(0.359)	(0.274)
Groups 2-5 students[b]	-0.931*	-0.859^	-1.011**
	(0.382)	(0.452)	(0.342)
Groups 6-10 students[b]	-0.583	-0.312	-0.874*
	(0.395)	(0.453)	(0.406)
Groups unknown size[b]	-0.464	-0.360	-0.585*
	(0.354)	(0.433)	(0.297)
Individual[b]	-0.398	-0.407	-0.395
	(0.381)	(0.476)	(0.331)
Individual and groups[b]	-0.603^	-0.469	-0.753*
	(0.358)	(0.432)	(0.314)
Unknown[b]	-0.529***	-0.507***	-0.548***
	(0.028)	(0.028)	(0.034)
Students without info[b,c]	-0.440	-0.320	-0.571^
	(0.354)	(0.430)	(0.302)
School year * Whole class	-0.089	-0.235	0.060
	(0.243)	(0.254)	(0.266)
School year * Groups 2-5	0.412^	0.371	0.453^
	(0.229)	(0.270)	(0.246)
School year * Groups 6-10	0.340	0.302	0.382
	(0.223)	(0.240)	(0.271)
School year * Groups unknown	0.067	0.059	0.076
	(0.161)	(0.209)	(0.184)
School year * Individual	0.490*	0.541*	0.434
	(0.235)	(0.263)	(0.279)
School year * Individual and groups	0.056	0.057	0.056
	(0.159)	(0.208)	(0.185)
School year * Unknown	0.000	-0.004	0.006
	(0.161)	(0.207)	(0.186)
School year * Without info	0.049**	0.052*	0.047*
	(0.018)	(0.022)	(0.022)
Student controls	Yes	Yes	Yes
School level controls	Yes	Yes	Yes
School-level fixed effects	Yes	Yes	Yes
Interaction effects of participation with other characteristics	Yes	Yes	Yes

Constant	-0.029	-0.173**	0.134^
	(0.063)	(0.062)	(0.071)
Observations	66,439	66,439	66,439
Clusters	456	456	456

Note: Robust standard errors in parentheses; *** p < 0.001, ** p < 0.01, * p < 0.05, ^ p < 0.1. [a] the reference category is the school year 2019/2020; [b] the reference category is students who did not participate in the remediation programs but are enrolled in schools that offer remediation programs. [c] Students who participate in remediation programs and for whom we do not have the questionnaire regarding the characteristics of the remediation program; this differs from the category ‘unknown’ as for these schools, we received the questionnaire; however, this specific question was not filled in (completely). Student controls include sex, migration background, parental education and income, and household structure; school-level controls include denomination, urbanization, and the disadvantage score of the school. Interaction effects of participation with other characteristics of remediation programs are organization, moment, goal, and type of support.
