# Supplementary material for: Effective remediation programs for vulnerable students to overcome learning loss
Source: PLoS One. 2025 May 14;20(5):e0323352. doi: 10.1371/journal.pone.0323352 (PMC12077795; doi:10.1371/journal.pone.0323352)
Supplement: S12 Table — (PDF) [file pone.0323352.s016.pdf]

**S12 Table. Effect of moment of the remediation program on students' achievements.**

| | Composite | Reading | Mathematics |
|---|---|---|---|
| School year 2020/2021[a] | 0.006 | 0.008 | 0.003 |
| | (0.009) | (0.009) | (0.010) |
| | | | |
| Program during regular hours[b] | -0.437 | -0.266 | -0.621* |
| | (0.323) | (0.381) | (0.287) |
| Program outside regular hours[b] | -0.756* | -0.641 | -0.881** |
| | (0.362) | (0.439) | (0.313) |
| Program during and outside regular hours[b] | -0.605^ | -0.481 | -0.738* |
| | (0.360) | (0.430) | (0.317) |
| Unknown[b] | -0.440 | -0.320 | -0.571^ |
| | (0.354) | (0.430) | (0.302) |
| Students without info[b,c] | -0.529*** | -0.507*** | -0.548*** |
| | (0.028) | (0.028) | (0.034) |
| School year * During regular hours | -0.180 | -0.215 | -0.143 |
| | (0.164) | (0.205) | (0.198) |
| School year * Outside regular hours | 0.049 | 0.011 | 0.088 |
| | (0.164) | (0.215) | (0.184) |
| School year * During and outside | -0.011 | -0.017 | -0.004 |
| | (0.176) | (0.216) | (0.204) |
| School year * Unknown | -0.000 | -0.004 | 0.006 |
| | (0.161) | (0.207) | (0.186) |
| School year * Students without info | 0.049** | 0.052* | 0.047* |
| | (0.018) | (0.022) | (0.022) |
| | | | |
| Student controls | Yes | Yes | Yes |
| School level controls | Yes | Yes | Yes |
| School-level fixed effects | Yes | Yes | Yes |
| Interaction effects of participation with other characteristics | Yes | Yes | Yes |
| | | | |
| Constant | -0.029 | -0.173** | 0.134^ |
| | (0.063) | (0.062) | (0.071) |
| | | | |
| Observations | 66,439 | 66,439 | 66,439 |
| Clusters | 456 | 456 | 456 |

Note: Robust standard errors in parentheses; *** p < 0.001, ** p < 0.01, * p < 0.05, ^ p < 0.1. [a] the reference category is the school year 2019/2020; [b] the reference category is students who did not participate in the remediation programs but are enrolled in schools that offer remediation programs. [c] Students who participate in remediation programs and for whom we do not have the questionnaire regarding the characteristics of the remediation program; this differs from the category 'unknown' as for these schools, we received the questionnaire; however, this specific question was not filled in (completely). Student controls include sex, migration background, parental education and income, and household structure; school-level controls include

denomination, urbanization, and the disadvantage score of the school. Interaction effects of participation with other characteristics of remediation programs are organization, group size, goal, and type of support.
