# Supplementary material for: Effective remediation programs for vulnerable students to overcome learning loss
Source: PLoS One. 2025 May 14;20(5):e0323352. doi: 10.1371/journal.pone.0323352 (PMC12077795; doi:10.1371/journal.pone.0323352)
Supplement: S13 Table — (PDF) [file pone.0323352.s017.pdf]

S13 Table. Effect of remediation program's organization on students' achievements.

	Composite	Reading	Mathematics
School year 2020/2021[a]	0.006	0.008	0.003
	(0.009)	(0.009)	(0.010)
Internal staff[b]	0.011	0.049	-0.037
	(0.345)	(0.420)	(0.292)
External staff[b]	0.389	0.490	0.278
	(0.400)	(0.455)	(0.391)
Internal and external staff[b]	0.035	0.058	0.005
	(0.355)	(0.435)	(0.299)
Unknown[b]	-0.440	-0.320	-0.571^
	(0.354)	(0.430)	(0.302)
Students without info[b,c]	-0.529***	-0.507***	-0.548***
	(0.028)	(0.028)	(0.034)
School year * Internal staff	-0.044	0.047	-0.129
	(0.166)	(0.195)	(0.189)
School year * External staff	-0.109	-0.091	-0.123
	(0.235)	(0.258)	(0.278)
School year * Internal and external staff	0.028	0.138	-0.079
	(0.181)	(0.212)	(0.204)
School year * Unknown	-0.000	-0.004	0.006
	(0.161)	(0.207)	(0.186)
School year * Students without info	0.049**	0.052*	0.047*
	(0.018)	(0.022)	(0.022)
Student controls	Yes	Yes	Yes
School level controls	Yes	Yes	Yes
School-level fixed effects	Yes	Yes	Yes
Interaction effects of participation with other characteristics	Yes	Yes	Yes
Constant	-0.029	-0.173**	0.134^
	(0.063)	(0.062)	(0.071)
Observations	66,439	66,439	66,439
Clusters	456	456	456

Note: Robust standard errors in parentheses; *** p < 0.001, ** p < 0.01, * p < 0.05, ^ p < 0.1. [a] the reference category is the school year 2019/2020; [b] the reference category is students who did not participate in the remediation programs but are enrolled in schools that offer remediation programs. [c] Students who participate in remediation programs and for whom we do not have the questionnaire regarding the characteristics of the remediation program; this differs from the category 'unknown' as for these schools, we received the questionnaire; however, this specific question was not filled in (completely). Student controls include sex, migration background, parental education and income, and household structure; school-level controls include

denomination, urbanization, and the disadvantage score of the school. Interaction effects of participation with other characteristics of remediation programs are moment, group size, goal, and type of support.
