# Supplementary material for: Effective remediation programs for vulnerable students to overcome learning loss
Source: PLoS One. 2025 May 14;20(5):e0323352. doi: 10.1371/journal.pone.0323352 (PMC12077795; doi:10.1371/journal.pone.0323352)
Supplement: S14 Table — (PDF) [file pone.0323352.s018.pdf]

S14 Table. Effect of remediation program's goal on students' achievements.

	Composite	Reading	Mathematics
School year 2020/2021[a]	0.006	0.008	0.003
	(0.009)	(0.009)	(0.010)
Language and math goal[b]	-1.056***	-0.909*	-1.197***
	(0.297)	(0.371)	(0.250)
Language goal[b]	-1.039***	-0.952**	-1.129***
	(0.282)	(0.348)	(0.252)
Math goal[b]	-1.227***	-1.033**	-1.425***
	(0.332)	(0.399)	(0.305)
Cognitive and non-cognitive goal[b]	-0.950**	-0.755*	-1.141***
	(0.289)	(0.365)	(0.242)
Non-cognitive goal[b]	-0.440	-0.320	-0.571^
	(0.354)	(0.430)	(0.302)
Students without info[b,c]	-0.529***	-0.507***	-0.548***
	(0.028)	(0.028)	(0.034)
School year * Language and math goal	0.219^	0.259	0.178
	(0.133)	(0.163)	(0.176)
School year * Language goal	0.193	0.260	0.125
	(0.130)	(0.165)	(0.172)
School year * Math goal	0.458*	0.492*	0.421^
	(0.193)	(0.215)	(0.229)
School year * Cognitive and non-cognitive goal	0.094	0.095	0.091
	(0.124)	(0.157)	(0.165)
School year * Non-cognitive goal	-0.000	-0.004	0.006
	(0.161)	(0.207)	(0.186)
School year * Students without info	0.049**	0.052*	0.047*
	(0.018)	(0.022)	(0.022)
Student controls	Yes	Yes	Yes
School level controls	Yes	Yes	Yes
School-level fixed effects	Yes	Yes	Yes
Interaction effects of participation with other characteristics	Yes	Yes	Yes
Constant	-0.029	-0.173**	0.134^
	(0.063)	(0.062)	(0.071)
Observations	66,439	66,439	66,439
Clusters	456	456	456

Note: Robust standard errors in parentheses; *** $p < 0.001$, ** $p < 0.01$, * $p < 0.05$, ^ $p < 0.1$. [a] the reference category is the school year 2019/2020; [b] the reference category is students who did not participate in the remediation programs but are enrolled in schools that offer remediation programs. [c] Students who participate in remediation programs and for whom we do not have the questionnaire regarding the characteristics of the remediation program; this differs from the category 'unknown' as for these schools, we received the questionnaire; however, this specific question was not filled in (completely). Student controls include sex, migration background, parental education and income, and household structure; school-level controls include denomination, urbanization, and the disadvantage score of the school. Interaction effects of participation with other characteristics of remediation programs are organization, group size, moment, and type of support.
