# Supplementary material for: Effective remediation programs for vulnerable students to overcome learning loss
Source: PLoS One. 2025 May 14;20(5):e0323352. doi: 10.1371/journal.pone.0323352 (PMC12077795; doi:10.1371/journal.pone.0323352)
Supplement: S15 Table — (PDF) [file pone.0323352.s019.pdf]

S15 Table. Effect of type of support on students' achievements.

	Composite	Reading	Mathematics
School year 2020/2021[a]	0.006	0.008	0.008
	(0.009)	(0.009)	(0.010)
Additional support and guidance[b]	-0.371	-0.260	-0.504*
	(0.242)	(0.268)	(0.223)
Purchase of new methods[b]	-0.364	-0.355	-0.392^
	(0.242)	(0.269)	(0.231)
Extended instructions[b]	-0.130	-0.104	-0.189
	(0.286)	(0.324)	(0.274)
Extended school days[b]	-0.046	0.075	-0.211
	(0.301)	(0.318)	(0.296)
Remedial teaching[b]	-0.868**	-0.740*	-1.019**
	(0.300)	(0.333)	(0.312)
Support during work[b]	-0.650*	-0.604*	-0.720*
	(0.284)	(0.304)	(0.289)
Unknown[b]	-0.245	-0.181	-0.339
	(0.275)	(0.301)	(0.260)
Students without info[b,c]	-0.529***	-0.507***	-0.548***
	(0.028)	(0.028)	(0.034)
School year * Additional support and guidance	-0.165	-0.315^	-0.003
	(0.144)	(0.183)	(0.146)
School year * Purchase of new methods	-0.176	-0.295	-0.044
	(0.155)	(0.190)	(0.168)
School year * Extended instructions	-0.153	-0.253	-0.039
	(0.173)	(0.226)	(0.173)
School year * Extended school days	-0.321	-0.410^	-0.217
	(0.198)	(0.235)	(0.206)
School year * Remedial teaching	0.028	0.047	0.015
	(0.217)	(0.309)	(0.183)
School year * Support durin work	-0.170	-0.290	-0.045
	(0.205)	(0.223)	(0.239)
School year * Unknown	-0.103	-0.226	0.032
	(0.167)	(0.202)	(0.175)
School year * Without info	0.049**	0.052*	0.046*
	(0.018)	(0.022)	(0.022)
Student controls	Yes	Yes	Yes
School level controls	Yes	Yes	Yes
School-level fixed effects	Yes	Yes	Yes
Interaction effects of participation with other characteristics	Yes	Yes	Yes

Constant	-0.029	-0.173**	-0.110***
	(0.063)	(0.062)	(0.026)
Observations	66,439	66,439	66,439
Clusters	456	456	456

Note: Robust standard errors in parentheses; *** p < 0.001, ** p < 0.01, * p < 0.05, ^ p < 0.1. [a] the reference category is the school year 2019/2020; [b] the reference category is students who did not participate in the remediation programs but are enrolled in schools that offer remediation programs. [c] Students who participate in remediation programs and for whom we do not have the questionnaire regarding the characteristics of the remediation program; this differs from the category ‘unknown’ as for these schools, we received the questionnaire; however, this specific question was not filled in (completely). Student controls include sex, migration background, parental education and income, and household structure; school-level controls include denomination, urbanization, and the disadvantage score of the school. Interaction effects of participation with other characteristics of remediation programs are organization, moment, goal, and group size.
