# Supplementary figures and images for: Effective remediation programs for vulnerable students to overcome learning loss
Source: PLoS One. 2025 May 14;20(5):e0323352. doi: 10.1371/journal.pone.0323352 (PMC12077795; doi:10.1371/journal.pone.0323352)

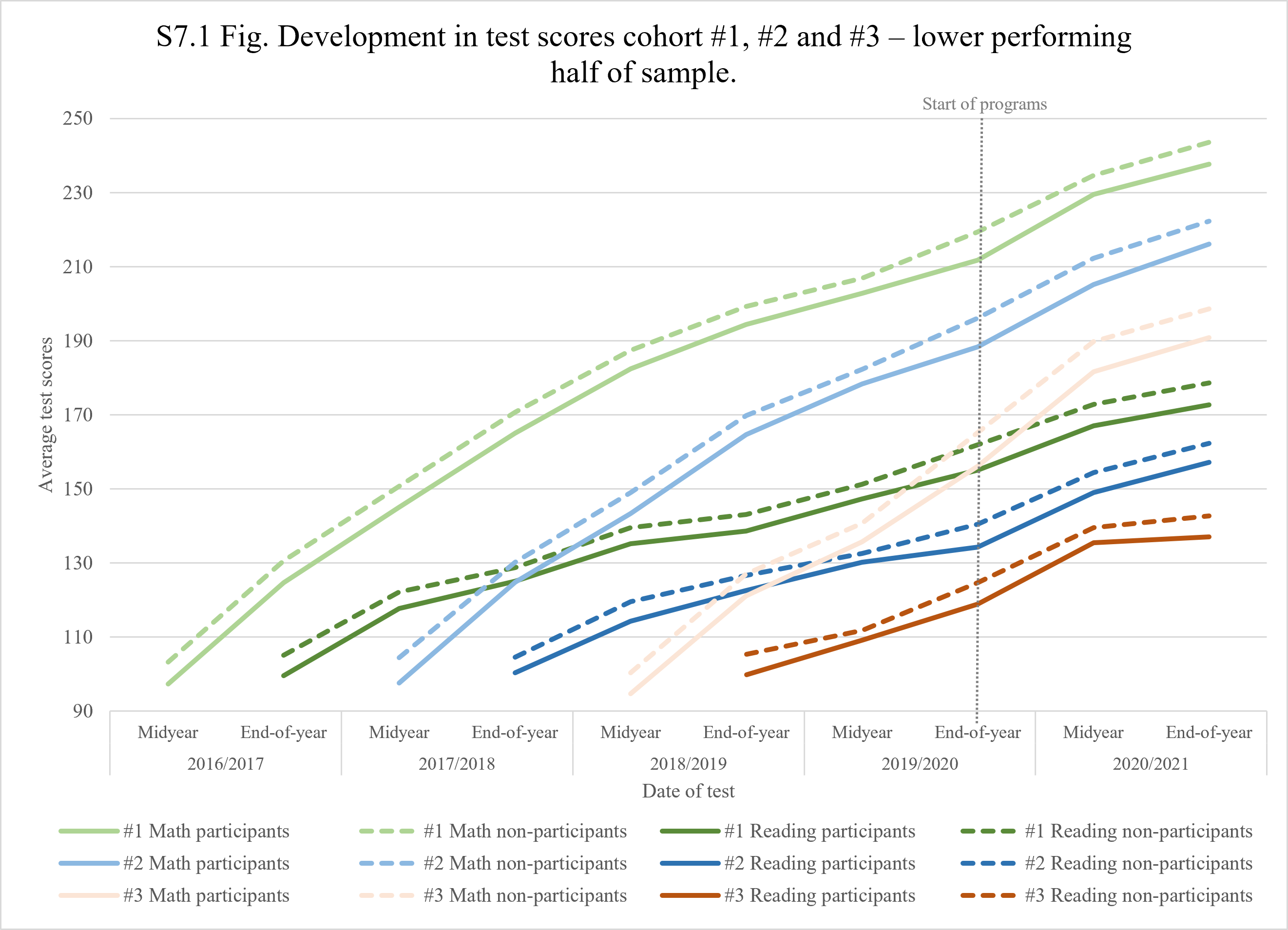

Supplement: S1 Fig — (PNG) [file pone.0323352.s020.png]

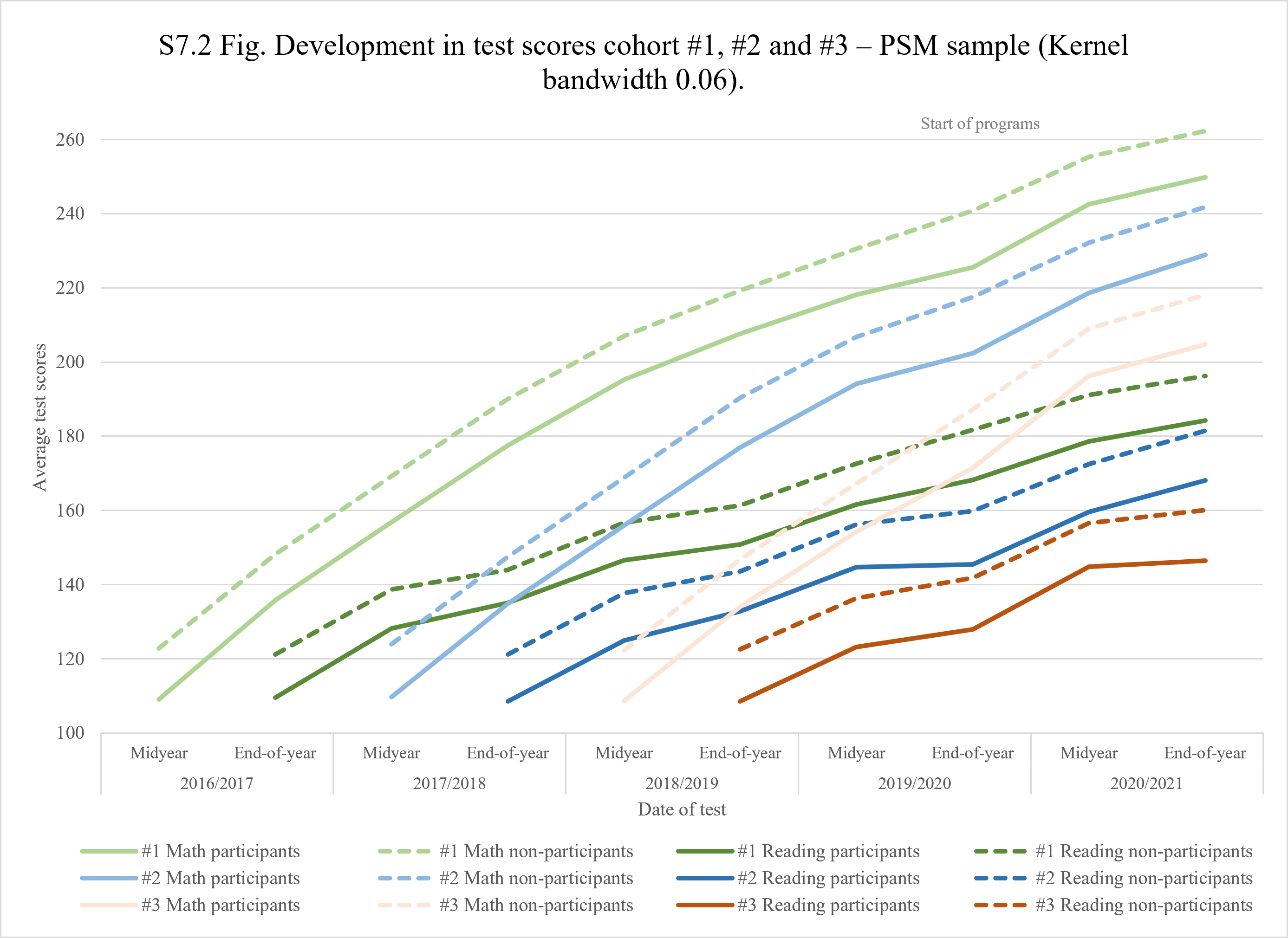

Supplement: S2 Fig — (PNG) [file pone.0323352.s021.png]
