# Supplementary material for: Effective remediation programs for vulnerable students to overcome learning loss
Source: PLoS One. 2025 May 14;20(5):e0323352. doi: 10.1371/journal.pone.0323352 (PMC12077795; doi:10.1371/journal.pone.0323352)
Supplement: Attachment — Submitted filename: Response to reviewers PLOSONE - Jacobs et al.pdf [file pone.0323352.s024.pdf]

Effective Remediation Programs for Vulnerable Students to Overcome Learning Loss

Response to reviewers

PLOSONE

February 2025

Thank you once again for your valuable feedback and suggestions. The reviewers' detailed comments and constructive criticism have significantly enhanced our paper, helping us to improve the paper's quality and clarity. We have carefully considered each comment and incorporated the necessary revisions. In the document below, we address all the points raised by both reviewers and the editor. We truly appreciate your time and effort.

Response to the Editor

Thank you for submitting a revision to your article, which engaged thoroughly with the points that reviewers raised. I asked both reviewers to look at your response and revised manuscript. This does lead me to ask for some further revisions, but I would like to explain the situation as I see it further. Dealing with the two reviewers in turn:

Reviewer 1 has changed their assessment to Minor Revisions. Their remaining comments are fairly general and, while you are welcome to take on their suggestions, I think you have essentially satisfied their suggestions and do not propose to send the revision that I am requesting to them.

Thank you for giving us another opportunity to revise our manuscript. We have indeed taken on some of reviewer 1's suggestions and responded to these below in our response to reviewer 1.

Reviewer 2 welcomes your strong engagement with their suggestions but, I think partly because of the improved clarity of the manuscript (which is, of course, welcome), has identified some additional concerns about aspects of the analyses for aims 2 and 3 and, so, retains a Major Revisions recommendation. I do share these concerns and, because our overriding concern should be to ensure the conclusions of the argument are well-supported by the analysis, also frame my request in this way.

Thank you. As for reviewer 2's remaining concerns, we have included detailed explanations in our response to reviewer 2. However, we also briefly summarize these below and address some points specifically regarding reviewer 2's feedback.

In respect of aim 2, the reviewer flags up alternative possibilities about the potential bias of the difference in differences estimator used. My read is that such potential bias make cause incorrect conclusions to be drawn from your analysis. As such, I am keen that this is addressed if possible. Specifically, I would encourage you to explore whether the matched difference-in-differences analysis suggested is possible with

the data that you have to hand (checking the plausibility of this strategy by whether you are then able to observe more encouraging parallel trends within the matched sample). However, mindful that it may not be possible with the data you have, you may alternatively submit a revision that much more clearly documents the potential biases that could arise such that the reader can very clearly judge for themselves the strength of evidence provided by this aspect of the study.

In response to the concern about potential biases in our results, we have explored the Difference-in-Differences analyses with propensity score matching (PSM). Furthermore, we have examined the results and pre-trends if we only keep the lower-performing half of our sample, which is the group of students on which the interventions were focusing. Based on the large similarities of these results, we have decided to include these as robustness checks in the manuscript. The results remain very robust when analyzing the DiD analyses with the PSM-matching and for the subsample of lower-performing students. Hence, we prefer the original results, as PSM-matching also imposes new assumptions and challenges, which we will elaborate on below. In the paper, we now have added an extra subsection titled "Robustness checks" in the "Results" section. We explain these two robustness checks in more detail below, in our response to reviewer 2.

In respect of aim 3, it seems to me (although, as always, you have the option to rebut this in your response) that it should be possible to address and revise this aspect of the manuscript based on the further explanations and suggestions provided by reviewer 2. I would, of course, also encourage you to take on board sundry suggestions provided by the reviewer (my summary here is not exhaustive).

We have looked into this and revised our analyses and manuscript now. We attempted to adjust our analyses based on reviewer 2's feedback to the best of our understanding in the previous round. However, based on the new feedback in the second round, we realize we apparently did not fully comprehend what reviewer 2 meant. In this round, we have once again attempted to include reviewer 2's suggestion to perform a school-level analysis, although we must confess that we still do not fully comprehend what reviewer 2 has in mind when suggesting this type of analysis. We have tried several ways to perform a school-level analysis, but unfortunately, we keep running into problems when we try to incorporate analyses at the school level or calculate effects at the school level, regardless of our approach.

We do agree with the underlying comment that it is problematic that the characteristics of the programs are interrelated, but the suggested solution to this problem of doing a school-level analysis does not seem feasible. Therefore, we have decided to proceed with the "mutually exclusive" variable approach, as also suggested by the reviewer. We explain this in more detail below, in our response to reviewer 2.

I would also like to thank you for engaging on the editorial points regarding financial disclosure and data availability. It is my assessment that these now meet the relevant policies (although the data are not publicly available you clearly document access procedures to be followed in gaining access, even if these may require moving to the Netherlands!).

Thank you again for engaging wholeheartedly in this process. I hope you will agree that it is helping to strengthen the robustness of the findings reported in this paper and, hence, their credibility. Although, of

course, I cannot guarantee acceptance of the manuscript at this stage, I do think it will be possible with the revisions requested.

Thank you! We hope our revisions are meeting your expectations!

Response to Reviewer 1

Dear Authors,
Thank you for addressing all the comments in the previous review. I have a few minor comments/suggestions this time.
You have three objectives in the introduction. I would recommend aligning these three objectives in:
(a) Literature review and theoretical framework (that needs your attention),
(b) Method (that you have done)
(c) Results (that you have done)
(d) Discussion (that needs your attention)
(e) Implication (that needs your attention).
I hope this alignment will create a clear flow of objective, process, and outcomes.

Thank you for suggesting an improved flow of the paper by better aligning the research aims of the paper in all sections of the paper. The research aims outlined in the methods section align with the three models we analyzed. Furthermore, in the results section, we have now incorporated headings to differentiate between the three aims of the paper. We have aligned the research questions in the results recap in the discussion and conclusion section. However, we have not incorporated this into the literature review and theoretical framework. This section mainly discusses literature and mechanisms related to the third research aim, discussing various remediation efforts. As we do not discuss a similar amount of literature regarding research aims 1 and 2, this section would be very much unbalanced. Hence, we decided not to use the suggested structure in this section.

Response to Reviewer 2

We especially want to thank reviewer 2 for taking the time to give such detailed and thoughtful comments. We have now incorporated these in the revised version of the paper. We hope you are satisfied with the changes that we have made.

As a short summary regarding your comments on research aim 2, based on your comments we ran additional analyses in which we conducted several PSM models, as well as reduced our sample to only the lower-performing half of our sample (which is the group of students that the interventions were focusing at) to better control for potential biases. However, we have decided to introduce these as robustness checks, as the results are very similar to our original model, as we will explain further below.

Regarding research aim 3, we have tried several ways to perform a school-level analysis, but unfortunately, we keep running into problems when we try to incorporate analyses at the school level or calculate effects at the school level, regardless of our approach. Hence, as per your suggestion, we have now created mutually exclusive variables with information about the remediation programs and used these in our analyses. A more detailed response to all your points can be found below.

<u>Research Aim 2</u>

(1.1) As noted in my initial review, the divergence in learning trends between the treatment and the control group before the intervention (seen in figures S7.2-4 in the SI and discussed in the existing literature on learning losses during COVID-19 in the Netherlands), means that the current diff-in-diff research design is not suited to provide a point estimate of the true size of the effect of the remedial education programs implemented in the school year 2020-21 (Research Aim 2). But while I was initially under the impression that the DiD estimator may (only) be downwardly biased (hence my comment on the lower-bound estimate), upon reading the new version of the manuscript, it became clear that it may indeed be biased in either direction. This becomes clear when we consider that any of following could be true, given the divergent pre-trends and the unique context of the COVID-19 pandemic right before the intervention:

- The DID-estimator may *underestimate* the true effect of the intervention, since the treatment group (which consists of lower achieving and socio-economically disadvantaged students) may have continued to have a lower learning rate than the control group (which consists of higher achieving and socio-economically advantaged students) in the absence of the intervention, perhaps because they continued to struggle with the consequences of the COVID-19 pandemic. This is what initially led me to proposed interpreting this as a 'lower-bound' estimate may suffice.
- But, (2) a second, (similarly) plausible scenario could be that the DID-estimator may *overestimate* the true effect of the intervention, since the treatment group may have recovered from the initial COVID-19 induced learning loss even in the absence of the intervention, for instance due to schools reopening in the Netherlands.

In short, due to the divergent pre-trends, the current DiD estimator could be biased in either direction, and the DiD research design does not allow for a meaningful inference on the true effect of the remedial learning interventions.

This leads me to the conclusion that the problem lies with the choice of comparison groups. The solution for approximating a more meaningful effect of the intervention may thus be to use a (propensity score) matching approach in order to compare students who are alike in terms of their previous attainment and socio-economic background, but who differ in whether they participated in the remedial education intervention. Given that the data at hand contain information on previous performance and socio-economic background this may be feasible. The question of course is how many low-performing students from low socio-economic backgrounds *did not* participate in any remedial learning intervention during the 2020-21 school year in the Netherlands. The feasibility of this alternative approach may be gauged from whether it allows for generating a control group that matches the pre-intervention learning trend during the 2019-20 school year of the treatment group. In other words, to correctly identify the effect of the remedial education intervention, one would need to work with a control group that incurred similar learning losses as the students in the treatment group, but did not receive remedial education.

Thank you for sharing your concerns regarding potential bias in the results of research aim 2. If we understand you correctly, your main concern regarding bias comes from the diverging pre-trends. Luckily, we do have sufficient variation in participation rate among the low-performing students from disadvantaged backgrounds. Hence, we have performed several additional analyses in which we create more balanced treatment and control groups, including your suggestion to use PSM models, with which we show very parallel pre-trends and confirm the robustness of our results. However, the methods of these additional analyses bring about new challenges, which is why we have opted to include these analyses as robustness checks rather than including (one of) them as our main analysis.

As for propensity score matching, this can indeed be used to create a more balanced treatment and control group. However, as PSM in itself ranks lower among the causality analysis methods one can employ, we have decided to combine the two techniques of DiD and PSM. The main weakness of PSM is that there might be a remaining part of the selection process due to unobservable characteristics. A DiD-model deals with this problem of unobservable background characteristics by using time-invariant fixed effects, which are similar before and after the policy introduction. Consequently, we have opted to match treatment and control groups using PSM and then generate a DiD estimator of the policy intervention. Although there are many ways to employ PSM, none come without challenges. Using Nearest-neighbor matching (1:1 or even 1:5) procedures severely reduces the sample size, which creates potential power issues. This might be problematic for research aim 2, especially for research aim 3, as we have to include many interaction terms and covariates in these regression models. And we strongly prefer to keep the same sample for all analyses in our paper. Therefore, we opted for Kernel matching, which allows us to include (almost) all observations within common support by applying Kernel weights in our regression. However, the downside of that is that we cannot use our original estimation method anymore in which we include school fixed effects (which are crucial as the interventions are organized at the school level), as varying weights per school are not allowed when using the fixed effect model (and in this case, we have varying weights per student).

We have also conducted an additional analysis to check for potential overestimation. Namely, we have conducted our main model with half the sample—the half of the students who scored lowest before the remediation programs. We also checked the parallel time trends for these students, which look more convincing. However, the downside of this method is that we lose many observations (including some of the

participants, which is highly undesirable), which is problematic, especially for answering research aim 3, as we explained above.

Therefore, we have added the two additional analyses as robustness checks in our paper, to better control for potential issues that may lead to over- or underestimation of main effect. A detailed and extensive description of these two robustness checks can be found below, after your comment on Figures S7.2-4.

I would like to emphasise that the issue of interpreting the DiD-estimator as a point estimate despite diverging pre-trends is rather serious. Many readers are likely to take the point estimate reported in the abstract (0.065 SD) at face value and conclude that the remediation programmes implement in the Netherlands after the COVID-19 pandemic were effective, even though the estimate may be strongly biased in either direction, as explained above. I hope that with the alternative approach suggested, it may be possible to address this issue. That said, if this issue cannot be addressed, it may not be possible to address the second research aim with the data at hand.

Thank you for voicing these concerns, you are absolutely right. We hope we have been able to address your concern regarding potential bias due to diverging pre-trends and differences between the treatment and control groups. However, based on your comment, we have also decided to nuance the use of the point estimate a bit. Although we have consistently found that the effect is roughly around 0.05 SD, based on our main models and the thorough robustness checks that we have conducted, we now nuance that a bit where we can.

In our conclusion and discussion, we take a closer look at the point estimate from our main model to put the positive impact of remediation programs into perspective, especially in comparison to the effect of regular education. Hence, we incorporated the point estimate into a simplified cost/benefit exercise. However, we have now included the point estimate and the confidence interval, emphasizing that there is some uncertainty around the point estimate. Furthermore, we have shortened this section in the conclusion and discussion to downsize the emphasis on this cost/benefit exercise.

The following paragraph has been adapted in the conclusion and discussion as follows: "Our Difference-in-Differences (DiD) analyses estimate the effect of remediation programs at approximately 0.05 SD, with a 95% confidence interval ranging from 0.019 to 0.085 SD. While this effect size may appear modest, it signals a meaningful and positive impact. Given the relatively short duration of these programs—typically between 25 and 40 hours—this gain is notable. To put this into perspective, the average yearly learning progress in primary education is about 0.4 SD under normal circumstances, a benchmark that was likely unattainable during the pandemic. Even within these constraints, remediation efforts contributed to students' progress. Considering the time investment, the observed effect suggests that remediation programs provided a positive return in terms of learning gains relative to the limited instructional hours. Therefore, these remediation programs are a solution to the COVID-19 related learning losses."

(1.2) It would be helpful to combine the first panel from figures S7.2-4 in one figure and include it in the main text (with the revised comparison groups, as proposed above). This would also help the reader gauge the substantive size of the effects. It is not clear, why Figure S7.1 is shown in the SI, given that this cohort

does not form part of the analysis. For the more detailed figures in the appendix, it would be important to harmonise the scale of the y-axis across the different panels.

Thank you for pointing this out. Indeed, this first cohort is not part of the analytical group; however, it has been shown to encompass the entire trajectory of one cohort throughout primary education. This cohort has now been excluded from the figures. Furthermore, we have now combined all these separate figures into one figure and have included it in the main text, as you suggested, as Figure 2. Additionally, while updating the pre-trend figures, we saw that we included the midyear and end-of-year tests of school year 2020/2021. As the end-of-year test of 2019/2020 and the end-of-year test of 2020/2021 are in our Difference-in-Differences, they should not be included in the pre-trends. Therefore, the pre-trend figures now do not include the test scores for cohort 1, and do not show the test scores for school year 2020/2021. The following figure below has been added to the manuscript as Figure 2, and to the Supplementary Information as Figure S7.1. In the figure the first cohort (green) started grade 1 of primary education in 2016/2017. The second cohort (blue) began in 2017/2018 in grade 1, and the third cohort (orange) started in 2018/2019. Therefore, all cohorts face the COVID-19 pandemic in different grade levels and participate in remediation programs in various grade levels. As reading does not have a midyear test in grade 1, the trends for reading start with the end-of-year test in grade 1.

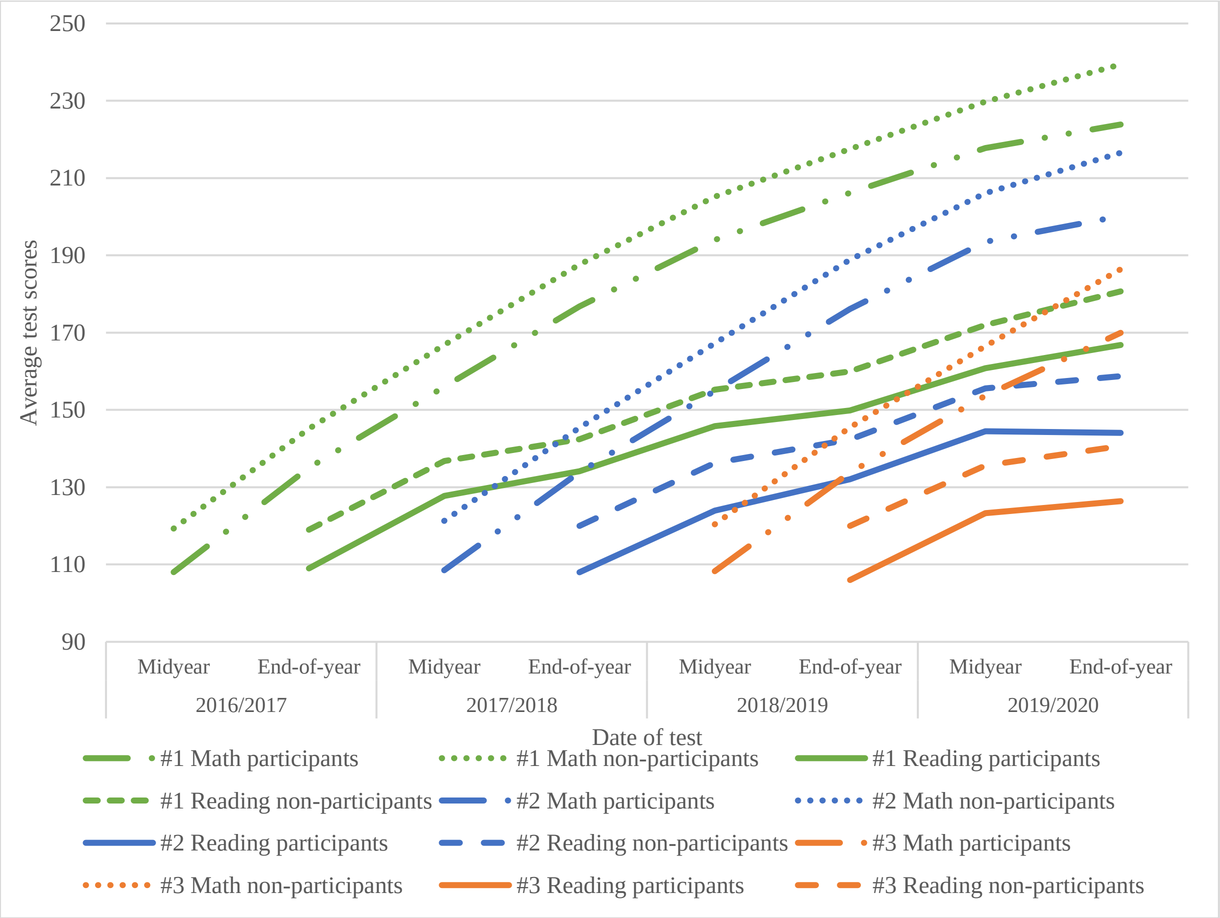

Figure 1. Pre-trends: Development in test scores cohorts #1, #2, and #3 – entire sample

Detailed explanation of our two robustness checks.

(1) *Propensity score matching analyses*

Our propensity score matching approach incorporates several student background characteristics to match participating and non-participating students. We use the students' test scores from the midyear test of the school year 2019/2020, right before the school closures due to COVID-19. Furthermore, we included several background characteristics of the students: gender, migration background, household structure, education level of the parents, the income of the parents, and lastly, the parents' working status.

As mentioned above, several matching procedures have their benefits and challenges. To explore matching as an alternative, we have decided to estimate several different PSM models, using these background characteristics, and compare them: (1) Kernel logit matching with different bandwidths [0.06 default; 0.02 and 0.12]; (2) 1:1 matching with replacement with different calipers [off, 0.10 and 0.25]; (3) 1:1 matching without replacement with different calipers [off, 0.10 and 0.25]; and (4) 1:5 matching with replacement with different calipers [off, 0.10 and 0.25].

In the first PSM option (1) we use Kernel matching, which allows us to weigh observations depending on their proximity to the treatment group based on all the background characteristics, with the main advantage that we hardly lose any students in our sample. The different bandwidth options allow for a higher or lower inclusion rate. This means that a larger bandwidth allows a greater distance between the matches, which leads to a larger inclusion of cases in the matching, although it also increases the risk of bias. The default setting in Stata is 0.06; we have included a somewhat more restricted value and a more lenient value. With this approach, we can use almost all students in our model, and these students receive a weight that can be considered in our Difference-in-Differences model.

The other three PSM options, options (2), (3), and (4), match specific cases to each other (nearest neighbor matching: NN-matching). This leads to a selection of students who have a match, meaning, based on background characteristics, we have found a similar "neighbor" in the treatment and control groups. This also entails that some students will not have a match based on our specifications, which leads to a (much) smaller sample. NN-matching consists of considering each treated (control) unit and searching for the control (treated) unit with the closest propensity score, i.e., the nearest neighbor. We use NN-matching 1:1 (which means that each treatment student is matched to one control student), and we use 1:5 (which means each treatment student is matched to a maximum of 5 control students, as there are way more control students than treatment students). Specifically, we estimate the following three PSM models: (i) 1:1 matching without replacement, (ii) 1:1 matching with replacement, and (iii) 1:5 matching with replacement. When we use the "replacement" option, a control-group student might be used more than once as a match for a treated student, whereas "without replacement" considers each student only once. We estimate these three models, each with three different caliper settings (no caliper, 0.10 caliper and 0.25 caliper). The caliper setting allows a maximum propensity score distance between the matched units. Sometimes, the best match for a specific case is still not a good match, as they are still quite different. These "bad matches" affect the treatment effect estimation if this is the case. The caliper setting, therefore, is used to determine the maximum distance allowed between the cases.

*So, we have 12 different PSM-model specifications (the four model specifications discussed below, each with three different bandwidth or caliper settings). Additionally, each matching procedure and model was estimated separately for reading, mathematics, and composite scores. This leaves us with 12 * 3 = 36 different PSM models to be estimated. These PSM models give all students weights, which can be added to the DiD analyses. It is important to note that with 1:1 or 1:5 matching, a large part of the sample does not receive a weight (as not all students find a match) and, therefore, will be left out of the specific sample and, hence, analysis.*

As mentioned before, we will use the weights gathered from the PSM models and incorporate these in our Difference-in-Differences analyses. The DiD model will then include a weight for all the different PSM models estimated separately for each domain. As a PSM weight cannot be used in the fixed effects model we use in our main model (with the xtreg package in Stata), as explained above, we employ a slightly different fixed effects approach where we cluster the standard errors at the school level and include a dummy for all schools in the sample. To compare the different matching models with the original results to check the robustness, it is important to first replicate the original results with this slightly different way of including fixed effects. Table 1 below shows the DiD-indicator differences between these approaches for the main model in the paper. Model 1 is the main model we use in the manuscript, where the DiD analyses are estimated using the xtregression command in Stata, which allows for clustering at the school level. The second model uses the normal regression command in Stata but additionally includes clustered standard errors at the school level (vce clusters) and incorporates dummies for all schools within the regression (which asks a lot more from the model, as it includes many more variables in the regression). As can be seen, the coefficients are very similar. The model presented in the paper, with the fixed effects DiD-analyses, shows point estimates for a composite of 0.052 SD, reading 0.050 SD and for mathematics, 0.054 SD. If we use a model where we include clustered standard errors and include a dummy for all schools, we find very similar point estimates for the DiD-estimator (the interaction effect), with only a slight difference for mathematics. For the models with the PSM weights, we use the second model and then additionally incorporate the weights for each model and each domain separately.

Table 1. Different Fixed-Effects (FE) approaches for the main model.

	Composite	Reading	Mathematics
(1) Main model as in manuscript (xtreg)	0.052**	0.050**	0.054**
(2) Model with clustered standard errors at school level and dummies for all schools (reg)	0.052**	0.050**	0.061**

Note: *** p < 0.001, ** p < 0.01, * p < 0.05, ^ p < 0.1.

Table 2 shows the results of the various PSM specifications for reading, mathematics, and the composite score separately. We see that for all models with the Kernel specification, the point estimates are very similar to those of the main model. Only for mathematics the point estimate is slightly smaller than before. Note that the sample size is slightly smaller, as we have around N = 66,000 in our main model and around N = 49,000 to N = 60,000 for the PSM models with Kernel matching.

The results for the nearest neighbor models are a bit mixed, where we especially see mixed results for the 1:1 with replacement mode, which is most likely due to power issues as compared to all other PSM models, this model has the lowest sample size (N = 8,600 to N = 10,913). The sample sizes of the 1:1 model without

replacement and the 1:5 model with replacement are slightly larger. All models, except the 1:1 with replacement model, show a similar point estimate for the DiD estimates around 0.040 SD to 0.050 SD. However, we often find insignificant relationships in the models with a smaller sample size. The kernel bandwidth models are the only models that do not suffer from sample size issues and, hence, are our preferred matching models. The effect sizes for the kernel bandwidth are similar compared to the main model's estimate.

Table 2. PSM-DiD results for all different PSM options.

	**Composite**	**Reading**	**Mathematics**
Kernel bandwidth = 0.06	0.049**	0.056**	0.043*
	N = 48,223	N = 48,858	N = 60,809
Kernel bandwidth = 0.02	0.047*	0.054*	0.042*
	N = 48,223	N = 48,858	N = 60,809
Kernel bandwidth = 0.12	0.052**	0.059**	0.046*
	N = 48,223	N = 48,858	N = 60,809
1:1 replacement	-0.011	-0.052	0.006
	N = 8,667	N = 8,792	N = 10,913
1:1 no replacement	0.045*	0.041^	0.050*
	N = 14,968	N = 15,183	N = 19,123
1:5 replacement	0.050	0.074^	0.051
	N = 12,669	N = 12,828	N = 15,586
1:1 replacement caliper = 0.25	-0.011	-0.052	0.006
	N = 8,667	N = 8,791	N = 10,913
1:1 no replacement caliper = 0.25	0.045*	0.041^	0.050*
	N = 14,968	N = 15,183	N = 19,123
1:5 replacement caliper = 0.25	0.050	0.074^	0.051
	N = 12,669	N = 12,823	N = 15,586
1:1 replacement caliper = 0.10	-0.011	-0.052	0.006
	N = 8,667	N = 8,791	N = 10,913
1:1 no replacement caliper = 0.10	0.045*	0.041^	0.050*
	N = 14,968	N = 15,183	N = 19,123
1:5 replacement caliper = 0.10	0.050	0.074^	0.051
	N = 12,669	N = 12,823	N = 15,586

Note: *** $p < 0.001$, ** $p < 0.01$, * $p < 0.05$, ^ $p < 0.1$.

As an additional analysis of the comparability of the matched treatment and control groups, we have analyzed the pre-trends again based on the matched samples. Figure 2 shows the pre-trends for the PSM sample with Kernel matching and bandwidth 0.06 (the middle option regarding bandwidth selection). Note that the pre-trends of the other Kernel bandwidths are very similar. The pre-trends show that between the midyear and end-of-year tests in the school year 2019/2020, the trends of participants and non-participants are quite similar. In Figure 2 the first cohort (green) started grade 1 of primary education in 2016/2017. The second cohort (blue) began in 2017/2018 in grade 1, and the third cohort (orange) started in 2018/2019. Therefore, all cohorts face the COVID-19 pandemic in different grade levels and participate in

remediation programs in various grade levels. As reading does not have a midyear test in grade 1, the trends for reading start with the end-of-year test in grade 1.

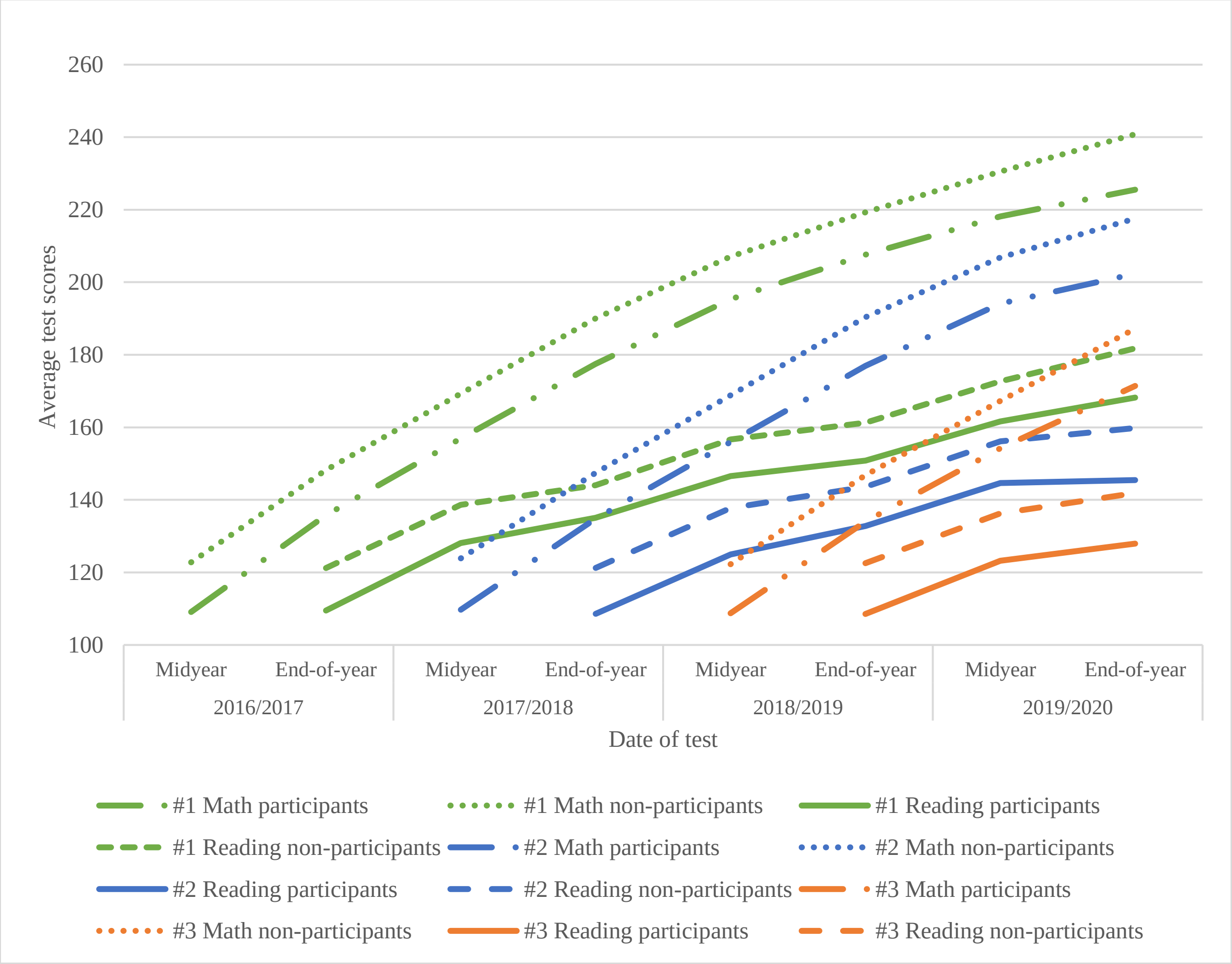

Figure 2. Pre-trends: Development in test scores cohorts #1, #2, and #3 – PSM sample (Kernel matching with bandwidth 0.06).

Based on all of the above, we have decided to incorporate the three Kernel matching models as robustness checks into the paper but not to include the other PSM matching options, as they significantly reduce the sample size, bringing about new challenges. We presented them in this response letter to show that we explored many possible ways to better align treatment and control groups. The robustness check is now incorporated in the main paper in the result section and added to the Supplementary Information section 5 for the models and section 7 for the pre-trends.

We have added the following text to the manuscript: "Lastly, we estimated our main models using Propensity Score Matching (PSM) approaches to align our control and treatment groups. Using the Kernel matching procedure allows us to weigh observations depending on their proximity to the treatment group based on background characteristics and previous test scores, with the main advantage that we hardly lose any students in our sample (more information about the PSM approach can be found in SI section 5.2). We

continue to use our DiD approach to analyze the effects of the remediation programs and include the PSM weights to better align our sample. The overall results confirm our main findings (see Supplementary Information Tables S5.7 – S5.10 for the full models).”

(2) <u>Difference-in-Differences for selection of the sample.</u>

As a second alternative approach, we have conducted our main estimation model again for only a selection of the sample. We divided our sample in half based on student's performance before the remediation programs (as it was specifically the lower-performing half of the students that was targeted with the remediation programs). We have conducted the main model again for the lowest 50% sample. The 50% sample consists of ~25.000 students. As the participants and non-participants of remediation programs in the lower-performing half of the sample are more alike, we wanted to check whether our findings are robust for this specification and check for potential biases, as suggested by the reviewer.

The results of this analysis can be found in Table 3 below (Table S5.6 in the Supplementary Information). The results show that the DiD effects for the composite and reading scores remain consistent with the main findings. However, now we do not find a significant effect anymore for mathematics, whereas, in the main model, we do have a significant effect. This could be due to power, although the point estimate also decreased quite a bit. Therefore, we have to treat our findings for mathematics with more caution, which we have now also emphasized in the paper.

In addition, we have also estimated the main DiD model with the smaller sample of lower-performing students using the kernel PSM-weights explained above. These results are comparable to what is shown in Table 3 below and can be found in the Supplementary Information as Table S5.10.

Table 3. DiD results for the average effect of remediation programs based on the lower-performing half of the sample.

	Composite	Reading	Mathematics
School year	0.029**	0.057***	0.062***
	(0.010)	(0.011)	(0.011)
Participants	-0.294***	-0.260***	-0.311***
	(0.015)	(0.017)	(0.017)
School year * participant	0.052**	0.043*	0.013
	(0.019)	(0.021)	(0.021)
Student controls	Yes	Yes	Yes
School level controls	Yes	Yes	Yes
School-level FE	Yes	Yes	Yes
Constant	-0.701***	-0.840***	-0.478**
	(0.168)	(0.190)	(0.180)
Observations	25,237	25,767	31,245
R-squared	0.071	0.056	0.075

Clusters	435	436	445

Note: Robust standard errors in parentheses; *** p < 0.001, ** p < 0.01, * p < 0.05, ^ p < 0.1. [a] the reference category is the school year 2019/2020; [b] the reference category is students who did not participate in the remediation programs but are enrolled in schools that offer remediation programs. Student controls include sex, migration background, parental education and income, and household structure, and school-level controls include denomination, urbanization, and the disadvantage score of the school.

For this specific sample, we have also calculated the pre-trends, as shown in Figure 3 below. These can also be found in the Supplementary Information Figure S7.2. These pre-trends show a more similar trend for participants and non-participants compared to the entire sample, with almost no diverging trends visible. During the school closures, we see no or less noticeable divergence in average test scores for the different cohorts. The first cohort (green) started grade 1 of primary education in 2016/2017. The second cohort (blue) began in 2017/2018 in grade 1, and the third cohort (orange) started in 2018/2019. Therefore, all cohorts face the COVID-19 pandemic in different grade levels and participate in remediation programs in various grade levels. As reading does not have a midyear test in grade 1, the trends for reading start with the end-of-year test in grade 1.

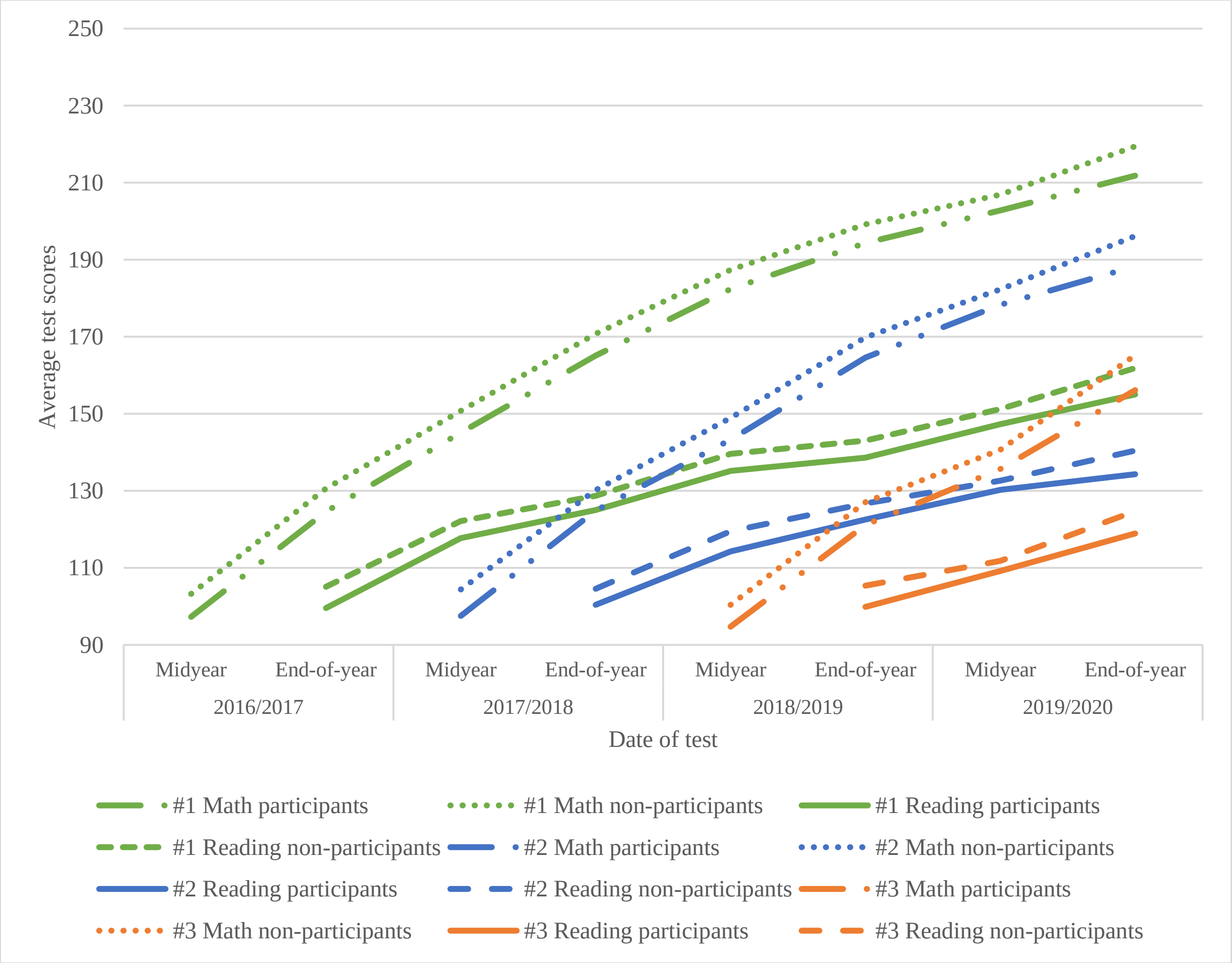

Figure 3. Pre-trends: Development in test scores cohorts #1, #2, and #3 – lower-performing half of the sample.

<u>Research Aim 3</u>

(2.1) There seems to be a misunderstanding concerning the alternative, school-level approach that I proposed for addressing the third research aim. In their reply to the reviewer comments, the author(s) note that they "extract[ed] average student scores at the school level for the 2020/2021 academic year when the remediation programs were implemented [...] and analys[ed] the content of the remediation programs with the extracted test scores for composite, reading and mathematics" (see reply to reviewer comments p.13). But my proposal was to extract the *effect sizes* of the remedial education program for each school in the sample (rather than the average student scores) and use these as the dependent variable with program characteristics as the focal independent variables, controlling for school characteristics and characteristics of the student body participating in the remedial education program. Effect sizes could be weighed according to the precision of each estimate to account for cross-school variation herein. If certain programme characteristics would lead to remediation programmes being more effective, one should see an association between these programme characteristics and the size of the effect of the remediation programme (even if the sample size is relatively small).

Conducting the analysis both at the school and at the individual level may be a possible way to test the robustness of the findings in response to the third research aim. In this respect, it is somewhat concerning that the results from the school-level analysis suggests that programmes run by 'internal staff' are more effective (see table R1 in the response letter to the reviewer report on p.15), while the results from the individual-level analysis (shown in Figure 2b) suggests that programmes run by 'external staff' are more effective. Such contradiction may be resolved by adjusting the school-level analysis as suggested.

Thank you for your elaborate explanation of your concerns with research aim 3. We had attempted to adjust our analyses based on your feedback to the best of our understanding in the previous round. However, based on the new feedback in the second round, we realize we apparently did not fully comprehend what you meant. In this round, we have once again attempted to include your suggestion to perform a school-level analysis, although we must confess that we still do not fully comprehend what you have in mind when suggesting this type of school-level analysis. In our view, any school-level approach is highly problematic due to the very small sample sizes per school, making reliable estimation impossible. However, we have tried estimating the effects in a separate analysis for each school, but the sample sizes per school are way too small and the coefficients between schools are not comparable because of school differences. Therefore, estimating the effect size per school does not seem to be feasible, and we are not familiar with any method to extract effect sizes per school from our main analyses, nor could we find suggestions in the literature on how to do this. (Perhaps the reviewer has a method in mind, but we unfortunately could not find any)

Given these challenges, we have decided to proceed with the alternative proposed by the reviewer—the mutually exclusive categories—which we now implement. We now better understand the reviewer's point through their proposal for mutually exclusive variables and fully agree that the previously applied approach was problematic. We have now implemented this new method in our paper and provide a detailed explanation below.

(2.2) As I noted in my initial report, there are two related problems concerning the individual-level analysis presented in Tables S6.1-14. The research question/aim focusses on examining "which types of remediation programs are most effective in enhancing student achievements" (p.4). In other words the aim is to compare the *relative* effectiveness of different types of remediation programmes. But the models shown in Tables S6.1-14 do not allow for inferences on the statistical significance of differences in the effectiveness between different programme types, since the reference category is students who did not participate in any remediation programme. The second problem is that the characteristics of the remedial education measures implemented in different schools are likely to co-vary and be confounded by characteristics that are not included in each of the separate models shown in Tables S6.1-13.

For their revised individual analysis, and in order to address the issue of confounding between programme characteristics, authors propose to "include these remediation program characteristics in our student-level difference-in-differences analysis". They also note that "our revised approach involves including all remediation program characteristics as control variables" (see reply to reviewer comments p.13). Since the relevant tables S6.1-14 in the SI only show 'yes' for the controls, it is not entirely clear what was done here, but it seems that only main effects were controlled for. This does not seem to solve the issue of confounding of the interaction between school-year and remediation type and also leaves the first problem noted above unsolved.

Aside from the school-level analysis proposed above, another way to address the second problem (to some extent) using an individual-level analysis might be to code the different types of remediation programmes into multiple, *mutually-exclusive* categories and run a two-way interaction between school year and this comprehensive, categorical measure of intervention type (with an additional category for no participation in any remedial program) and select one intervention type (perhaps the most common one), as the base category.

Thank you for suggesting this alternative approach. As mentioned above, we have decided to go ahead with this approach, as we do not see how we can make the school-level analysis work. Before we explain the different variables with mutually exclusive categories that we have created based on the content of the remediation program, we would like to provide more detail about how we received the information regarding the content, to be transparent about the steps we are taking to get to the new variables with mutually exclusive categories. Schools were required to complete a questionnaire detailing their proposed remediation program to secure the funds for the remediation programs. Based on this questionnaire, we received information about the organization and content of the remediation programs. There are several questions in this questionnaire we used for these new variables. This information has now also been added to the Supplementary Information section 1.5.2.

1. *Question 1: The first question focused on the program's content and included a drop-down menu with predefined options, allowing schools to select as many as were applicable. The question asked, "What type of program are you planning to offer?" with options including the purchase of additional teaching materials for monitoring, additional support and guidance, sports/games/culture, behaviour and communication, parental involvement, expanding digital education, creating successful experiences, developing study strategies, and summer or holiday school. If a school selected "Other, namely," a text box immediately appeared for them to specify the type of support they intended to provide.*

2. *Question 2: Additionally, if a school selected "Additional support and guidance," a follow-up question appeared with a second drop-down menu, offering further details about the specific support measures the school could offer. These options included extended school days, extended or additional instruction, remedial teaching in small groups, guided practice and automatization, pre-teaching, additional individual support, homework guidance, and extra support during independent work.*
3. *Question 3: Schools were asked to provide insight into when the remediation efforts would take place. They could indicate whether the program occurred during regular school hours, outside of regular school hours, or during and outside of regular school hours.*
4. *Question 4: Schools needed to indicate the size of the remediation program and the size of the groups receiving additional support. This could vary from entire classrooms to individual guidance. Schools could choose from the following options: whole class/grade, small groups of two to five students, small groups of six to ten students, groups of unknown size, individual support, individual support as well as in small groups, or unknown.*
5. *Question 5: The remediation programs were established to enhance skills or achieve specific objectives. The following goals were outlined: mathematics goal, language goal, general school performance goal, social-emotional goal, study skills goal, teacher professionalization goal, sports goal, and a cultural/excursions/creative goal.*
6. *Question 6: Supervisors guide and assist students throughout the remediation program. Different supervisors are available depending on whether schools collaborate with other institutions or if the program is outsourced. Supervisors may be internal, external, or a mix of both. Schools also had to distinguish between qualified and unqualified supervisors. Additionally, internal supervisors may consist of teachers, educational support staff, or teaching assistants. However, this question was filled in quite poorly, with many missing data. Therefore, this distinction is not made in our new variable.*
7. *Question 7: Schools were also requested to list potential collaborations with other institutions. These external collaborations could include partnerships with other schools, businesses, cultural institutions, municipalities, educational consultancy firms, sports associations, or teacher training colleges. Schools should indicate whether they solely work with their internal professionals, fully rely on external professionals from these other organizations, or use a combination of both.*

Drawing from the information provided in the questionnaire, we performed exploratory factor analyses to establish more structure and overlap between these variables. These factors offered initial insights into the clustering of several variables. Based on the information from this questionnaire, we identified five variables that we wanted to include in the analyses. These categories within these variables are mutually exclusive between schools within their own variable but not across different variables (we will present correlations between the variables below). Furthermore, categories such as "unknown," "missing," or "other" are often included among these variables, as schools did not fill in all questions of the questionnaire, and we do not want to drop schools because of missing information on one variable. We operationalize the five variables for our analyses regarding the characteristics of remediation programs as follows:

- *<u>Moment of the program:</u> Schools can set up the remediation program during different times, namely '1' remediation program offered during school, '2' remediation program offered after school, '3'*

remediation program offered during and after school, ‘4’ unknown, where the school has not answered this question. Note that the regular school day hours in the Netherlands are from 8:30/9:00 to 15:00/15:30.

- *Organization of the program: Both internal and external parties can help organize the remediation program. This variable captures the organization of the remediation program based on three aspects: the supervisors, executors, and outsourcers. These three variables are combined to create a new variable, the new organization variable consists of ‘1’ only internal staff (such as teachers, assistants, interns), ‘2’ only external staff, ‘3’ a combination of internal and external staff, or ‘4’ unknown, when the school has not provided any detail on the organization of their remediation program.*
- *Goal of the program: The goals the remediation program wanted to reach can be clustered into ‘1’ cognitive goals (math and language goals), ‘2’ language goals, ‘3’ mathematic goals ‘4’ cognitive and non-cognitive goals, or ‘5’ non-cognitive goals (socio-emotional or study skills).*
- *Group size: The group size variable consists of the number of students per group for whom the remediation program is offered simultaneously. The variable distinguishes between ‘1’ for the entire class, ‘2’ for groups with 2 to 5 students, ‘3’ for groups with 6 to 10 students, ‘4’ groups with an unknown number of students, ‘5’ individual programs, ‘6’ individual and group support, and ‘7’ unknown, where the school has not answered this question.*
- *Type of support offered during the program: this is a variable based on two questions in the questionnaire: first, the “what” and second, the “if extra support and guidance, then:”. This variable, type of support, is constructed based on answers of the two questions. To create a variable that categorizes the types of support offered, we looked at what types of support are offered, how many schools chose for certain types of support and whether they are offered in combination with other types of support or not. For the first question of the questionnaire, concerning “what” was offered, schools mostly offer “additional teaching materials” or “additional support and guidance”. For the second question, the follow up question regarding which extra support and guidance is offered, schools often chose “extended instructions”, “extended schooldays”, “additional support during independent work”, or “remedial teaching”, or combinations of these. Additionally, many schools answered “other” or left the entire second question open. Based upon these insights, we have created the following categorization: ‘1’ additional support and guidance [without a further specification of what this additional support entails], ‘2’ purchase of new methods [these new methods are mainly monitoring tools for teachers], ‘3’ extended instructions [including pre-teaching and guided automation and practice], ‘4’ extended school day [including homework supervision], ’5’ remedial teaching [including individual help], ‘6’ support during individual work, ‘7’ other [often interventions that only a small fraction of the schools chose, for example, summer schools or sport and cultural programs], or ‘8’ unknown/not filled in.*

Based on this, we have estimated a DiD-model which includes all interactions between the treatment and the five variables regarding the characteristics of the remediation programs simultaneously in the model, while controlling for student and school-level variables, with school-level FE and clustered standard errors. In addition, we have checked the correlations between the characteristics of remediation programs to make sure there is no large overlap between them. The results of the correlation analyses show that some categories across these five variables have a correlation of around 0.5 maximum, while categories such as

“unknown” across the variables have correlations around 0.7. Based on this, we decided to include all interaction terms in our preferred model.

Results of the characteristics of remediation programs

Tables 4 to 8 below present the results regarding the characteristics of the remediation programs. They can also be found in the Supplementary Information as Tables S6.1 – S6.5. The results are discussed in the manuscript more elaborately, below a shorter summary of the results can be found.

We find that the timing and organization of remediation programs are not related to the learning growth of participants in remediation programs. The group size, program goal(s) and type of support show significant relationships with learning growth.

The group size of remediation programs has a significant influence on student’s learning growth. The results show that students in individual remediation programs have significant positive increases in their test scores for reading comprehension compared to non-participants. For mathematics, on the other hand, programs in small groups, approximately between 2 and 5 students, have positive effects on test scores compared to non-participants. For the composite score, we find positive effects for programs in both small groups and individually.

In general, programs that have set goals to improve the cognitive skills of their students show significant positive results. Some programs aim to improve language and numeracy skills, whereas others focus on either. For students’ composite scores, we see that programs with language and mathematical goals also show positive significant effects. For programs that have only set a goal for mathematical skills, we find positive and significant effects on both students' language and mathematics test scores.

Finally, the type of support offered to students in the programs is examined. Here, we do not find many significant results, suggesting that the specific type of support provided may be less important than simply ensuring that students receive additional support. However, for reading, we identify two significant negative relationships compared to students not in remediation programs.

Additional thoughts regarding the results

The findings once more indicate a strong relation between mathematics goals and improved reading test scores among students. We share the reviewer’s view that this is a counter-intuitive result. However, we have performed many checks to verify its accuracy, and the results consistently show the same outcomes, also when applying the new approach.

Table 4. Effect of remediation program’s group size on students’ achievements (Table S6.1).

	Composite	Reading	Mathematics
School year 2020/2021[a]	0.006	0.008	0.003
	(0.009)	(0.009)	(0.010)
Whole class[b]	-0.035	-0.021	-0.072
	(0.307)	(0.359)	(0.274)
Groups 2-5 students[b]	-0.931*	-0.859^	-1.011**
	(0.382)	(0.452)	(0.342)

Groups 6-10 students[b]	-0.583	-0.312	-0.874*
	(0.395)	(0.453)	(0.406)
Groups unknown size[b]	-0.464	-0.360	-0.585*
	(0.354)	(0.433)	(0.297)
Individual[b]	-0.398	-0.407	-0.395
	(0.381)	(0.476)	(0.331)
Individual and groups[b]	-0.603^	-0.469	-0.753*
	(0.358)	(0.432)	(0.314)
Unknown[b]	-0.529***	-0.507***	-0.548***
	(0.028)	(0.028)	(0.034)
Students without info[b,c]	-0.440	-0.320	-0.571^
	(0.354)	(0.430)	(0.302)
School year * Whole class	-0.089	-0.235	0.060
	(0.243)	(0.254)	(0.266)
School year * Groups 2-5	0.412^	0.371	0.453^
	(0.229)	(0.270)	(0.246)
School year * Groups 6-10	0.340	0.302	0.382
	(0.223)	(0.240)	(0.271)
School year * Groups unknown	0.067	0.059	0.076
	(0.161)	(0.209)	(0.184)
School year * Individual	0.490*	0.541*	0.434
	(0.235)	(0.263)	(0.279)
School year * Individual and groups	0.056	0.057	0.056
	(0.159)	(0.208)	(0.185)
School year * Unknown	0.000	-0.004	0.006
	(0.161)	(0.207)	(0.186)
School year * Without info	0.049**	0.052*	0.047*
	(0.018)	(0.022)	(0.022)
Student controls	Yes	Yes	Yes
School level controls	Yes	Yes	Yes
School-level fixed effects	Yes	Yes	Yes
Interaction effects of participation with other characteristics	Yes	Yes	Yes
Constant	-0.029	-0.173**	0.134^
	(0.063)	(0.062)	(0.071)
Observations	66,439	66,439	66,439
Clusters	456	456	456

Note: Robust standard errors in parentheses; *** p < 0.001, ** p < 0.01, * p < 0.05, ^ p < 0.1. [a] the reference category is the school year 2019/2020; [b] the reference category is students who did not participate in the remediation programs but are enrolled in schools that offer remediation programs. [c] Students who participate in remediation programs and for whom we do not have the questionnaire regarding the content of the remediation program; this differs from the category 'unknown' as for these

schools, we received the questionnaire; however, this specific question was not filled in (completely). Student controls include sex, migration background, parental education and income, and household structure; school-level controls include denomination, urbanization, and the disadvantage score of the school. Interaction effects of participation with other characteristics of remediation programs are organization, moment, goal, and type of support.

Table 5. Effect of moment of the remediation program on students' achievements (Table S6.2).

	**Composite**	**Reading**	**Mathematics**
School year 2020/2021[a]	0.006	0.008	0.003
	(0.009)	(0.009)	(0.010)
Program during regular hours[b]	-0.437	-0.266	-0.621*
	(0.323)	(0.381)	(0.287)
Program outside regular hours[b]	-0.756*	-0.641	-0.881**
	(0.362)	(0.439)	(0.313)
Program during and outside regular hours[b]	-0.605^	-0.481	-0.738*
	(0.360)	(0.430)	(0.317)
Unknown[b]	-0.440	-0.320	-0.571^
	(0.354)	(0.430)	(0.302)
Students without info[b,c]	-0.529***	-0.507***	-0.548***
	(0.028)	(0.028)	(0.034)
School year * During regular hours	-0.180	-0.215	-0.143
	(0.164)	(0.205)	(0.198)
School year * Outside regular hours	0.049	0.011	0.088
	(0.164)	(0.215)	(0.184)
School year * During and outside	-0.011	-0.017	-0.004
	(0.176)	(0.216)	(0.204)
School year * Unknown	-0.000	-0.004	0.006
	(0.161)	(0.207)	(0.186)
School year * Students without info	0.049**	0.052*	0.047*
	(0.018)	(0.022)	(0.022)
Student controls	Yes	Yes	Yes
School level controls	Yes	Yes	Yes
School-level fixed effects	Yes	Yes	Yes
Interaction effects of participation with other characteristics	Yes	Yes	Yes
Constant	-0.029	-0.173**	0.134^
	(0.063)	(0.062)	(0.071)
Observations	66,439	66,439	66,439
Clusters	456	456	456

Note: Robust standard errors in parentheses; *** p < 0.001, ** p < 0.01, * p < 0.05, ^ p < 0.1. [a] the reference category is the school year 2019/2020; [b] the reference category is students who did not participate in the remediation programs but are enrolled

in schools that offer remediation programs. [c] Students who participate in remediation programs and for whom we do not have the questionnaire regarding the content of the remediation program; this differs from the category ‘unknown’ as for these schools, we received the questionnaire; however, this specific question was not filled in (completely). Student controls include sex, migration background, parental education and income, and household structure; school-level controls include denomination, urbanization, and the disadvantage score of the school. Interaction effects of participation with other characteristics of remediation programs are organization, group size, goal, and type of support.

Table 6. Effect of remediation program’s organization on students’ achievements (Table S6.3).

	Composite	Reading	Mathematics
School year 2020/2021[a]	0.006	0.008	0.003
	(0.009)	(0.009)	(0.010)
Internal staff[b]	0.011	0.049	-0.037
	(0.345)	(0.420)	(0.292)
External staff[b]	0.389	0.490	0.278
	(0.400)	(0.455)	(0.391)
Internal and external staff[b]	0.035	0.058	0.005
	(0.355)	(0.435)	(0.299)
Unknown[b]	-0.440	-0.320	-0.571^
	(0.354)	(0.430)	(0.302)
Students without info[b,c]	-0.529***	-0.507***	-0.548***
	(0.028)	(0.028)	(0.034)
School year * Internal staff	-0.044	0.047	-0.129
	(0.166)	(0.195)	(0.189)
School year * External staff	-0.109	-0.091	-0.123
	(0.235)	(0.258)	(0.278)
School year * Internal and external staff	0.028	0.138	-0.079
	(0.181)	(0.212)	(0.204)
School year * Unknown	-0.000	-0.004	0.006
	(0.161)	(0.207)	(0.186)
School year * Students without info	0.049**	0.052*	0.047*
	(0.018)	(0.022)	(0.022)
Student controls	Yes	Yes	Yes
School level controls	Yes	Yes	Yes
School-level fixed effects	Yes	Yes	Yes
Interaction effects of participation with other characteristics	Yes	Yes	Yes
Constant	-0.029	-0.173**	0.134^
	(0.063)	(0.062)	(0.071)
Observations	66,439	66,439	66,439
Clusters	456	456	456

Note: Robust standard errors in parentheses; *** p < 0.001, ** p < 0.01, * p < 0.05, ^ p < 0.1. [a] the reference category is the school year 2019/2020; [b] the reference category is students who did not participate in the remediation programs but are enrolled in schools that offer remediation programs. [c] Students who participate in remediation programs and for whom we do not have the questionnaire regarding the content of the remediation program; this differs from the category 'unknown' as for these schools, we received the questionnaire; however, this specific question was not filled in (completely). Student controls include sex, migration background, parental education and income, and household structure; school-level controls include denomination, urbanization, and the disadvantage score of the school. Interaction effects of participation with other characteristics of remediation programs are moment, group size, goal, and type of support.

Table 7. Effect of remediation program's goal on students' achievements (Table S6.4).

	Composite	Reading	Mathematics
School year 2020/2021[a]	0.006	0.008	0.003
	(0.009)	(0.009)	(0.010)
Language and math goal[b]	-1.056***	-0.909*	-1.197***
	(0.297)	(0.371)	(0.250)
Language goal[b]	-1.039***	-0.952**	-1.129***
	(0.282)	(0.348)	(0.252)
Math goal[b]	-1.227***	-1.033**	-1.425***
	(0.332)	(0.399)	(0.305)
Cognitive and non-cognitive goal[b]	-0.950**	-0.755*	-1.141***
	(0.289)	(0.365)	(0.242)
Non-cognitive goal[b]	-0.440	-0.320	-0.571^
	(0.354)	(0.430)	(0.302)
Students without info[b,c]	-0.529***	-0.507***	-0.548***
	(0.028)	(0.028)	(0.034)
School year * Language and math goal	0.219^	0.259	0.178
	(0.133)	(0.163)	(0.176)
School year * Language goal	0.193	0.260	0.125
	(0.130)	(0.165)	(0.172)
School year * Math goal	0.458*	0.492*	0.421^
	(0.193)	(0.215)	(0.229)
School year * Cognitive and non-cognitive goal	0.094	0.095	0.091
	(0.124)	(0.157)	(0.165)
School year * Non-cognitive goal	-0.000	-0.004	0.006
	(0.161)	(0.207)	(0.186)
School year * Students without info	0.049**	0.052*	0.047*
	(0.018)	(0.022)	(0.022)
Student controls	Yes	Yes	Yes
School level controls	Yes	Yes	Yes
School-level fixed effects	Yes	Yes	Yes
Interaction effects of participation with other characteristics	Yes	Yes	Yes

Constant	-0.029	-0.173**	0.134^
	(0.063)	(0.062)	(0.071)
Observations	66,439	66,439	66,439
Clusters	456	456	456

Note: Robust standard errors in parentheses; *** p < 0.001, ** p < 0.01, * p < 0.05, ^ p < 0.1. [a] the reference category is the school year 2019/2020; [b] the reference category is students who did not participate in the remediation programs but are enrolled in schools that offer remediation programs. [c] Students who participate in remediation programs and for whom we do not have the questionnaire regarding the content of the remediation program; this differs from the category 'unknown' as for these schools, we received the questionnaire; however, this specific question was not filled in (completely). Student controls include sex, migration background, parental education and income, and household structure; school-level controls include denomination, urbanization, and the disadvantage score of the school. Interaction effects of participation with other characteristics of remediation programs are organization, group size, moment, and type of support.

Table 8. Effect of type of support on students' achievements (Table S6.5).

	**Composite**	**Reading**	**Mathematics**
School year 2020/2021[a]	0.006	0.008	0.008
	(0.009)	(0.009)	(0.010)
Additional support and guidance[b]	-0.371	-0.260	-0.504*
	(0.242)	(0.268)	(0.223)
Purchase of new methods[b]	-0.364	-0.355	-0.392^
	(0.242)	(0.269)	(0.231)
Extended instructions[b]	-0.130	-0.104	-0.189
	(0.286)	(0.324)	(0.274)
Extended school days[b]	-0.046	0.075	-0.211
	(0.301)	(0.318)	(0.296)
Remedial teaching[b]	-0.868**	-0.740*	-1.019**
	(0.300)	(0.333)	(0.312)
Support during work[b]	-0.650*	-0.604*	-0.720*
	(0.284)	(0.304)	(0.289)
Unknown[b]	-0.245	-0.181	-0.339
	(0.275)	(0.301)	(0.260)
Students without info[b,c]	-0.529***	-0.507***	-0.548***
	(0.028)	(0.028)	(0.034)
School year * Additional support and guidance	-0.165	-0.315^	-0.003
	(0.144)	(0.183)	(0.146)
School year * Purchase of new methods	-0.176	-0.295	-0.044
	(0.155)	(0.190)	(0.168)
School year * Extended instructions	-0.153	-0.253	-0.039
	(0.173)	(0.226)	(0.173)
School year * Extended school days	-0.321	-0.410^	-0.217

	(0.198)	(0.235)	(0.206)
School year * Remedial teaching	0.028	0.047	0.015
	(0.217)	(0.309)	(0.183)
School year * Support durin work	-0.170	-0.290	-0.045
	(0.205)	(0.223)	(0.239)
School year * Unknown	-0.103	-0.226	0.032
	(0.167)	(0.202)	(0.175)
School year * Without info	0.049**	0.052*	0.046*
	(0.018)	(0.022)	(0.022)
Student controls	Yes	Yes	Yes
School level controls	Yes	Yes	Yes
School-level fixed effects	Yes	Yes	Yes
Interaction effects of participation with other characteristics	Yes	Yes	Yes
Constant	-0.029	-0.173**	-0.110***
	(0.063)	(0.062)	(0.026)
Observations	66,439	66,439	66,439
Clusters	456	456	456

Note: Robust standard errors in parentheses; *** p < 0.001, ** p < 0.01, * p < 0.05, ^ p < 0.1. [a] the reference category is the school year 2019/2020; [b] the reference category is students who did not participate in the remediation programs but are enrolled in schools that offer remediation programs. [c] Students who participate in remediation programs and for whom we do not have the questionnaire regarding the content of the remediation program; this differs from the category 'unknown' as for these schools, we received the questionnaire; however, this specific question was not filled in (completely). Student controls include sex, migration background, parental education and income, and household structure; school-level controls include denomination, urbanization, and the disadvantage score of the school. Interaction effects of participation with other characteristics of remediation programs are organization, moment, goal, and group size.

Of all the information relating to research aim 3 that we have elaborated on above, we have included the following details in the manuscript: (i) operationalization and descriptive statistics in the section "Data and methods" concerning these five new variables with mutually exclusive categories have been added; (ii) the equation for estimating the third research question now incorporates the interaction of all five variables with participation; (iii) the Results section "Content of remediation programs" has been rewritten in accordance with the new results; (iv) in the Results section "Robustness checks', now includes additional analyses regarding the characteristics of the remediation programs.

Aside from the manuscript itself, we have included additional information concerning the characteristics of the remediation programs, along with the new analyses of research aim 3, in the Supplementary Information. We have added (i) more detailed information about how the data was collected regarding the characteristics of the programs (see Supplementary Information Section 1.5 and 3.5); (ii) operationalization and descriptive statistics (see Supplementary Information Section 3.5); (iii) the results of all DiD analyses (see Supplementary Information Section 6); (iv) the results of robustness checks (see Supplementary Information Section 6).

(2.3) It seems problematic, that the manuscript only selects the statistically significant results from Tables S6.1-14 for inclusion in the main text (in Figures 2a and 2b). Readers may not appreciate the possible multiple-testing bias when only seeing two out of the fourteen models that were tested in the main text. Using either of the school-level or the individual-level approach indicated above, may be one way to present the results for the different program characteristics in a simple yet encompassing way. It would be helpful to show all results from this analysis in one coefficient plot.

In the previous version, we analyzed various characteristics of the remediation programs, which made portraying all these effects in one figure or table rather unclear. Consequently, we decided to concentrate on the few elements that demonstrated promising results, while clearly stating in the accompanying text that we also encountered numerous non-significant results. Although this was never our intention, we do understand that this might come across as undesirable. With the new variables with mutually exclusive categories, we reduced the characteristics of remediation programs to five different variables, and these are now all showcased in the manuscript.

(2.4) It would be helpful to explain more clearly what is meant by the concepts used to describe the different program characteristics (e.g. what is meant by ‘remedial teaching’ or 'focus on language'), as well as how they are operationalised and measured. Relatedly, it would be helpful to add more theoretical discussion of why the characteristics of different remedial education measures that are examined are thought to be relevant.

Thank you for this suggestion. Based on this, we now have reorganized the literature review, where we discuss effective interventions from previous studies to highlight and explain the interventions we are examining more carefully. Furthermore, the information about the questionnaire, as added above, as well as included in the Supplementary Information, aims to explain these concepts more thoroughly as well. We hope this makes the characteristics of remediation programs more clear to the reader.

<u>Reviewer 2 other points</u>

(3.1) I agree that the current way to portray predicted probabilities in Figure 1a is similarly hard (or even harder) to interpret as the original figure using odds ratios. Using average marginal effects may allow for portraying the association btw. programme participation and different factors in a more parsimonious way while also allowing for the interpretation of the substantive size of the effect. Relatedly, it would be helpful to interpret the substantive size of the effects of different factors shown in Figure 1a on programme participation. The text currently only seems to comment on the statistical significance and the sign of coefficients, but not on their substantive size.

Thank you for your feedback regarding the figures. Indeed, the text could further elaborate on the effect sizes. This has now been added to the manuscript.

“The results indicate that students with less educated parents are more likely to participate in remediation programs (1.5% more likely than the mean), whereas students with both parents in employment are less

likely to participate (1% less likely than the mean). There is also a strong inverse relationship between test scores and participation in remediation programs. Students with extremely low test scores (-6 SD) have the highest probability of participation (90%), while those with average (0 SD, 15%) or above average scores (3 SD, 2%; 4 SD, 1%) are considerably less likely to participate. This pattern suggests that students with lower test scores are more inclined to participate, with the probability decreasing sharply as scores increase. This implies that lower educational achievement is an important indicator for participation."

(3.2) Some important labels are missing in figures (e.g. y-axis labels in Figures 1a and 1b).

Thank you; these have been added now.
