# Supplementary material for: Effective remediation programs for vulnerable students to overcome learning loss
Source: PLoS One. 2025 May 14;20(5):e0323352. doi: 10.1371/journal.pone.0323352 (PMC12077795; doi:10.1371/journal.pone.0323352)
Supplement: Attachment — Submitted filename: Response_to_reviewers_PLOSONE_-_Jacobs_et_al_auresp_3.pdf [file pone.0323352.s025.pdf]

**Effective Remediation Programs for Vulnerable Students to Overcome Learning Loss**

*Response to reviewers*

*PLOSONE*

*April 2025*

---

*Thank you once again for the opportunity to revise the paper based on the editor's and reviewer's comments. We are grateful to both the editor and the reviewer for their valuable feedback, which we have incorporated into this revised version to the best of our ability. We hope it meets the expectations. In this document, we provide a detailed explanation of the changes and additions made in response to the feedback provided.*

**Response to the Editor**

Thank you for submitting your manuscript to PLOS ONE. After careful consideration, we feel that it has merit but does not fully meet PLOS ONE's publication criteria as it currently stands. Therefore, we invite you to submit a revised version of the manuscript that addresses the points raised during the review process. As discussed in the previous round, I have only sought a review from one of the previous reviewers so we are just ensuring that the important issues they raise have been dealt with as best as possible. Indeed, I would anticipate being able to accept the paper if you make the minor revisions noted without recourse to further review.

*Thank you for giving us another opportunity to revise our manuscript.*

Ultimately, I think the biggest residual issue comes down to a disagreement over whether the parallel trends assumption is supported by the evidence provided in Figure 2, with the reviewer continuing to express concern in this regard. Looking closely at the final two points of Figure 2 I must agree that there is visible evidence of widening in more than one of the cohort/subject combinations, albeit not sizeable. Divergence in trends just ahead of treatment is, of course, potentially a particular concern because it may suggest something about the selection process into treatment being associated with pupils' trajectories.

*Thank you for highlighting this primary concern. We understand the concerns regarding the parallel trends and the differences between participating and non-participating students. The issues this raises about selection into treatment due to the widening gaps are, on one hand, a concern, and on the other hand, a confirmation of the right selection for the treatment group. Clearly, as schools were only permitted to establish the remediation programs for a small portion of their students, the selection was focused on those most in need of these programs. As research aim 1 shows, schools determine which students are 'most in need' of additional hours and support based on the test scores immediately preceding the implementation of the remediation programs. This aligns with the findings of research aim 1 and Figure 1, as well as the parallel trends, which show similarities up to the initial school closures due to COVID-19.*

I do agree, therefore, that it is important to caveat the findings with the risks of bias that may result from a lack of parallel trends. Ultimately, this is important regardless of the quality of pre-trends which are only suggestive since the (untestable) identifying assumption of difference in differences is that there would have been parallel trends between the treatment and comparison groups in the absence of treatment.

*Indeed, the untestable identifying assumption regarding the parallel trends, had the remediation programs not taken place, is an interesting point that has been raised. One could argue that, prior to the COVID-19 pandemic, the parallel trends were so similar that, had the treatment not occurred, the widening gaps would have persisted, as they had not been decreasing previously. However, we do not know this for sure and the parallel time trend figures are not as strong as we hoped they would be. Therefore, we have added an additional explanation to the section discussing parallel trends to mention the risk of bias due to widening gaps in the pre-trends. Below, we provide a detailed response to all additions and changes made to the figure and the manuscript.*

I, therefore, ask that you do: note these (small) differences in trends just ahead of treatment in your discussion of Figure 2 and the risks that these pose for potential biases. In support of this, I support the reviewer's suggestion to reinstate the 2020/21 data into Figure 2 to help readers judge the change in gradients involved both pre- and post-treatment. I suggest including a vertical line between the pre-test and post-test periods to avoid any ambiguity. I would also say that while I like having this information in a single reference figure, following so many different patterns is a bit challenging — I would suggest more use of colours and just two patterns for the lines, perhaps variants of the same colour across Reading and Maths.

With these additions, readers will then be able to judge for themselves the quality of the evidence presented when you lay it out clearly for them with the strengths and risks.

*Thank you. Let us start with the figure. We have adjusted the parallel trend figures in accordance with your suggestion, including the year 2020/2021 and the vertical line to indicate pre-trends and post-trends. Additionally, the colours and patterns have been adjusted. A solid line now always represents the participating students, and a dashed line always represents non-participating students. Cohort 1 is green, cohort 2 is blue and cohort 3 is orange. Reading always has the darker colour and mathematics has the lighter colour. Please find the updated Figure below.*

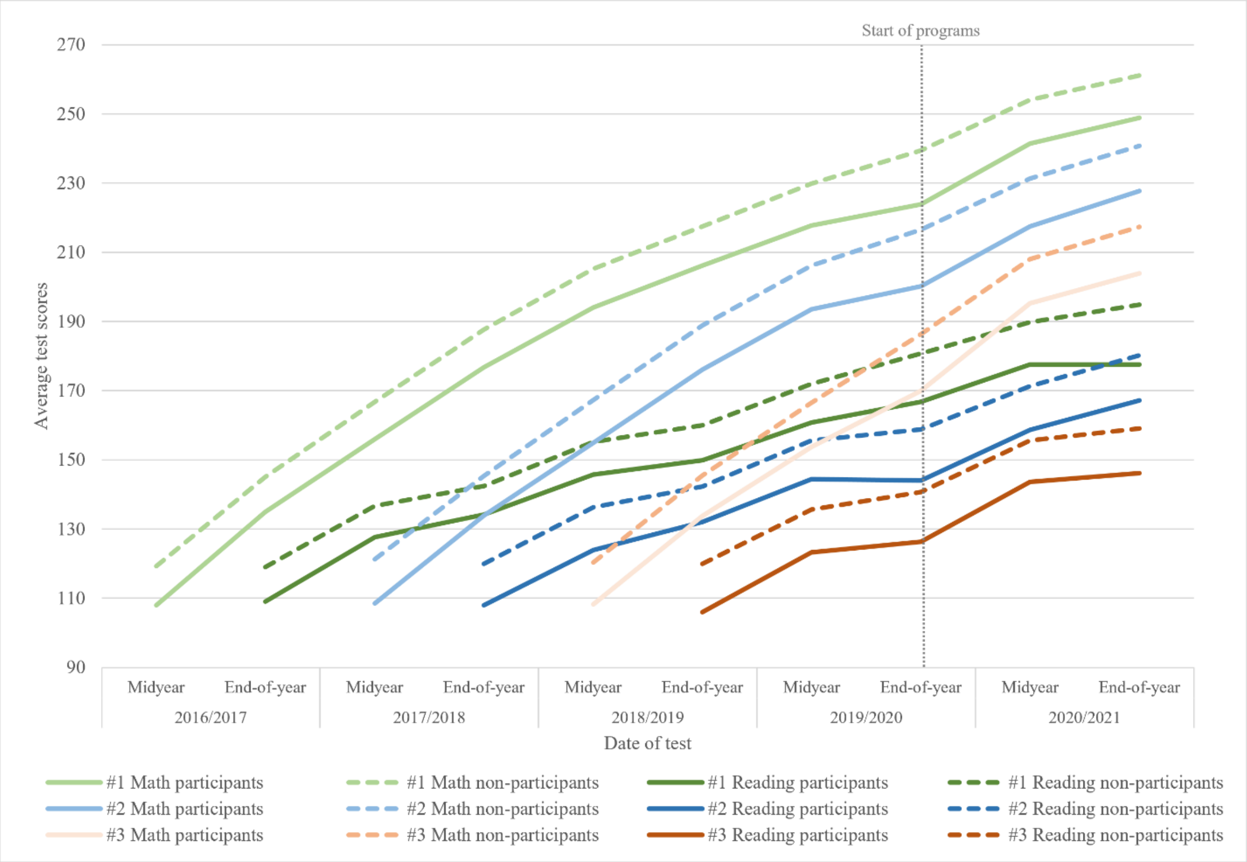


**Fig 2: Development of test-scores for the different cohorts in the sample.**

*This fig shows the parallel time trends for participants and non-participants in mathematics and reading for the three cohorts used in our analyses both before and after the remediation programs. The fig shows the pre-trends up until the end-of-year test in 2019/2020 and the post-trends in school year 2020/2021. The vertical line in the fig represents the transition from pre-trends to post-trends. The first cohort (green) started grade 1 of primary education in 2016/2017. The second cohort (blue) began in 2017/2018, and the third cohort (orange) started in 2018/2019. As reading does not have a midyear test in grade 1, the trends for reading start with the end-of-year test in grade 1.*

*Accompanying this figure, we discuss the pre-trends on page 18. This is a partially new paragraph, as some sentences were already present in the main text, however, we now elaborate more on the divergence in the pre-trends.*

*The manuscript now includes the following text: "The crucial assumption of the DiD analysis is the parallel time trend assumption (48). Fig 2 displays the time trends for the cohorts of students we include in our analyses. The results in Fig 1 have already shown that selection into the treatment is not random, rather that lower educational achievement is an important indicator for participation. In the pre-trends in Fig 2, we see divergence in progress between students who participated in the programs and non-participating students, reflecting the obvious differences in early learning losses after the first school closures, making*

*it important to consider that the overall effects of the program might be biased in either direction (see Supporting Information section 7 for the parallel time trends).''*

*Additionally, the parallel trend figures for the PSM sample and half of the sample (Supporting Information Figures S7.2 and S7.3) have been updated accordingly. These are discussed in the Robustness Checks section of the manuscript, and a separate reference to these two figures is provided in the Supporting Information.*

The point made regarding the programme characteristics analyses being appropriately caveated is also important to make in this similar spirit.

*Thank you. We have added this in the discussion section of the manuscript. In this section, we briefly touched upon the diverging pre-trends in the manuscript, but this section has now been updated. The following paragraph can be found in the discussion now: ''Given the divergence in the pre-trends regarding test score progress between the midyear test of 2019/2020 and end-of-year test in 2019/2020 among participating and non-participating students, it is important to consider that the overall effects of the program might be biased in either direction. The estimate may be biased upward if the participating students would have recovered without the remediation programs, such as due to the reopening of schools in general. Conversely, it may be biased downward if disadvantaged students, who were more likely to participate, would have continued to experience lower learning growth without the support of remediation programs. Interpreting the results in this paper as a causal effect of the remediation programs requires caution. However, this approach aims to approximate causality as good as possible, considering that these programs have a non-random treatment group. While it remains possible that our estimates are subject to both upward and downward bias, we have taken the necessary steps to strengthen their robustness. Overall, the evidence indicates that remediation programs were effective, though the precise magnitude of the effect may vary depending on the chosen specification. The same caution is needed when interpreting the estimates regarding the different characteristics of remediation programs as these estimates may be subject to bias or may be confounded by unobserved heterogeneity. Our robustness checks, including PSM models and analyses focusing on the lower-performing half of students, show somewhat more comparable pre-trends. The results of these robustness checks are largely consistent with our main outcomes.''*

**Journal requirements: reference list**

Please review your reference list to ensure that it is complete and correct. If you have cited papers that have been retracted, please include the rationale for doing so in the manuscript text, or remove these references and replace them with relevant current references. Any changes to the reference list should be mentioned in the rebuttal letter that accompanies your revised manuscript. If you need to cite a retracted article, indicate the article's retracted status in the References list and also include a citation and full reference for the retraction notice.

*In the current revision, no changes have been made to the manuscript's reference list. All references mentioned remain unchanged, and the reference list is complete and up to date.*

**Response to Reviewer 2**

Reviewer #2: Thank you for this revised version of the manuscript. I appreciate all of the work that the authors have done to expand on the analyses of the initial manuscript. I also appreciate that authors engage so carefully with the feedback provided. Below I focus on an assessment of the key elements of the analysis in the current version of the manuscript: (1) the DID analysis of the overall treatment effect of the remedial learning program, and (2) the analysis of how program effects co-vary with the characteristics of specific remedial education measures chosen by different schools.

*We would like to thank reviewer 2 once again for taking the time to engage with this paper and for the insightful comments provided. We hope you are satisfied with the changes we have made.*

The DID analysis with both the matched sample and the subsample of low-performing students are certainly informative. However, it is clear from Figures 1-3 shown in the response to the reviewer comments, that the comparison groups are (a) not balanced on performance and, more importantly, (b) there continues to be a divergence in the pre trend between the mid-year test and the end-of-year test. I recognize that authors may feel like they have reached the end of what they can do with the data at hand and would not want to run further analyses to identify a comparison group that follows a similar the pre-trend, as the treatment group. If that is the case, I believe that it is important for the authors to be clearer about the limitations of their DID. Somewhat surprisingly, the current version of the manuscript still states that "the crucial assumption of the DiD analysis, the parallel time trend assumption, is checked and met" (p.18), when it is evidently not met. Similarly, Section of the SI still asserts that "the parallel time trend shows that both participants and non-participants move parallel over time in their developing test scores. This crucial identifying restriction is checked and shows that this assumption is not violated" (p.46). It would be important to correct this and to write more clearly when interpreting their results (p.18 onwards) that (a) the parallel trends assumption does *not* hold, (b) that results may be biased in either direction, and (c) that the effects shown should therefore be interpreted with utmost caution and further research is needed to properly identify the causal effect of the remedial intervention.

*Thank you for pointing this out. Indeed, the sentence on page 18 of the manuscript and the one on page 46 in the Supporting Information should not have been phrased so strongly. This has now been revised. Additionally, the caution regarding the interpretation of the results due to the divergent trends has been addressed in the discussion of Figure 2. We have included the following text: "The crucial assumption of the DiD analysis is the parallel time trend assumption (48). Fig 2 displays the time trends for the cohorts of students we include in our analyses. The results in Fig 1 have already shown that selection into the treatment is not random, rather that lower educational achievement is an important indicator for participation. In the pre-trends in Fig 2, we see divergence in progress between students who participated in the programs and non-participating students, reflecting the obvious differences in early learning losses after the first school closures, making it important to consider that the overall effects of the program might be biased in either direction (see Supporting Information section 7 for the parallel time trends)."*

*Furthermore, the discussion includes a separate paragraph that specifically addresses this issue. Please find the paragraph below, following the other feedback provided.*

In my view it would be important to extend the timeline shown in Figures 1-3 in the response to the reviewer report (i.e. Figure 2 in the main text, and Figures S7.1-3 in the SI) to include the 2020/2021 mid-year test and the 2020/2021 end-of-year test. This would allow the reader to visually assess the substantive significance of both the pre-trend divergence and the treatment effect. Of course this would mean that these figures should be renamed from showing only 'pre-trends' to showing 'over-time change in the performance of the comparison groups'. Relatedly, while authors state that Figure 3 (in the response letter) shows "almost no diverging trends", this is of course a matter of interpretation, and showing how the performance of the comparison group continues to develop in the the 2020/2021 mid-year test and the 2020/2021 end-of-year test would allow the reader to gauge the substantive size of both the divergence in the pre-trend and the post-intervention trend.

*Thank you for your suggestions and feedback regarding Figure 2 (parallel time trends) and the recommendation to extend it. We have adjusted the parallel trend figures accordingly, including the years 2020/2021, and have added a vertical line to indicate the transition from pre-trends to post-trends. Furthermore, the colours and patterns have been modified to enhance the visibility of the different lines in the figure. In the figure, a solid line represents participating students, while a dashed line represents non-participating students. Cohort 1 is green, cohort 2 is blue, and cohort 3 is orange. Reading is depicted in the darker colour variant, while mathematics is represented in the lighter colour variant. Please find the updated figure below and in the manuscript.*

The analyses of different program characteristics has improved as a result of the new approach of categorising and analysing these program characteristics. That said, here too, it would be important for the manuscript to highlight more clearly in the interpretation of these results (p.20-23) that the results shown constitute descriptive (as opposed to causal) evidence, that the shown associations may be confounded by unobserved heterogeneity, and that results should therefore should be interpreted with caution.

*Thank you. Indeed, the caution required is necessary for both the main analyses conducted and the analyses regarding the characteristics of the programs. In the conclusion section of the manuscript, we have created a separate paragraph that discusses the pre-trends and their implications for the estimates presented in this study. Some of the content in this paragraph was already present in the discussion; however, by establishing it as a separate paragraph and adding further explanation, we hope to enhance clarity.*

*The following paragraph can now be found in the conclusion: "Given the divergence in the pre-trends regarding test score progress between the midyear test of 2019/2020 and end-of-year test in 2019/2020 among participating and non-participating students, it is important to consider that the overall effects of the program might be biased in either direction. The estimate may be biased upward if the participating students would have recovered without the remediation programs, such as due to the reopening of schools in general. Conversely, it may be biased downward if disadvantaged students, who were more likely to participate, would have continued to experience lower learning growth without the support of remediation programs. Interpreting the results in this paper as a causal effect of the remediation programs requires caution. However, this approach aims to approximate causality as best as possible, considering that these programs have a non-random treatment group. While it remains possible that our estimates are subject to both upward and downward bias, we have taken the necessary steps to strengthen their robustness. Overall, the evidence indicates that remediation programs were effective, though the precise magnitude of the effect*

*may vary depending on the chosen specification. The same caution is needed when interpreting the estimates regarding the different characteristics of remediation programs as these estimates may be subject to bias or may be confounded by unobserved heterogeneity. Our robustness checks, including PSM models and analyses focusing on the lower-performing half of students, show somewhat more comparable pre-trends. The results of these robustness checks are largely consistent with our main outcomes.''*
