# Supplementary material for: Effective remediation programs for vulnerable students to overcome learning loss
Source: PLoS One. 2025 May 14;20(5):e0323352. doi: 10.1371/journal.pone.0323352 (PMC12077795; doi:10.1371/journal.pone.0323352)
Supplement: Attachment — Submitted filename: Response to reviewers PLOSONE - Jacobs et al.docx [file pone.0323352.s023.docx]

**Effective Remediation Programs for Vulnerable Students to Overcome Learning Loss**

*PLOSONE*

*October 2024*

*Thank you very much for all your valuable feedback and suggestions. Thanks to all the detailed comments and constructive criticism, we were able to significantly improve our paper. The insights have helped us to improve the quality and clarity of our work. We have carefully considered each of the comments and incorporated them into the revision of the paper. In the document below, we specifically address all points raised by both reviewers and the editor. Thank you for your time and effort.*

**Response to main points mentioned by academic editor**

*Thank you for providing us with the opportunity to revise the paper. We want to specifically address points 1 and 4 you raised, related to the data availability statement and the literature section of the paper. Points 2 and 3 will be addressed in the response to reviewers 1 and 2.*

1. I have some concerns in relation to your current data availability statement (also noted by Reviewer 2 in their selection of the 'No' option in relation to the data availability review question) and its compatibility with the journal's stated policy in this area. While I am sympathetic to the fact that not all data will be shareable due to legal or ethical concerns, PLOS' policy requires you to be considerably more specific with the information you provide on with whom readers can make contact about the possibility of accessing the data. For example, the statement "Our access was granted through a partnership with a non-profit organization that established special agreements to facilitate this research. Researchers seeking access to the same data must engage in a similar partnership arrangement." needs to be amended to provide the details required by the data availability policy section on acceptable restrictions in relation to third-party data on this page: https://journals.plos.org/plosone/s/data-availability

*Thank you for pointing at this. We have now revised the Data Availability Statement and expanded the information provided about the data. The updated version can be found below and in the cover letter. We hope this provides all the necessary information for data accessibility and replication purposes. In addition, we have included the ethical approval for data collection in the manuscript.*

*“This section contains information on the data collection for the study "The Effects of Remediation Programs during the COVID-19 Pandemic on the Achievements of Vulnerable Students". The study is based on data from the Netherlands Cohort Study on Education (NCO in Dutch) and information about remediation programs sourced from schools, including participation lists and content details.*

*Access to the NCO data is only possible via the Remote Access (RA) infrastructure of Statistics Netherlands. The data cannot be shared publicly because of privacy restrictions and cannot be exported from the secure virtual environment at Statistics Netherlands. However, all data are available at Statistics Netherlands, and researchers can request data access provided they work at an institution with which Statistics Netherlands has agreements. Requests for data access can be sent to info@nationaalcohortonderzoek.nl. A detailed description of the data and the access procedure can be found in Haelermans, C., T. Huijgen, M. Jacobs, M. Levels, R. van der Velden, L. van Vugt and S. van Wetten (2020). Using Data to Advance Educational Research, Policy and Practice: Design, Content and Research Potential of the Netherlands Cohort Study on Education. European Sociological Review, 36(4), 643-662, https://doi.org/10.1093/esr/jcaa027.*

*However, data on remediation programs are not openly requestable for researchers. Data obtained from funding recipients and participation lists obtained from schools are confidential and cannot be shared due to legal and ethical restrictions. Researchers seeking access for replication purposes may contact the authors and can create a project at Statistics Netherlands (provided they work at an institution with which Statistics Netherlands has agreements). The authors are allowed to provide the data on the remediation programs under strict conditions within other projects at Statistics Netherlands.”*

4. Both reviewers suggest the potential the strengthen the conceptual discussion of programme characteristics and the potential benefit of using a theoretical framework to discuss their importance.

*Both reviewers highlighted the need to strengthen the literature section. Reviewer 1, in remarks 4, 5, and 7, emphasised the importance of clarifying the effectiveness of interventions, specifying their target areas, and expanding the discussion on learning loss and various remediation efforts. Reviewer 2 suggested that the reported effect sizes for each intervention should be clarified and interpreted. In response to these comments, we have thoroughly reviewed and revised the entire literature section. Below, we provide a brief overview of the changes made, with more detailed responses to the reviewers' points provided separately. We give a short summary here: First, we have elaborated more on the literature related to the school closures and the learning losses, referencing several review studies that all concluded negative effects during the school closures, with worse effects for disadvantaged students. Second, for all interventions, we have elaborated on effect sizes per domain (reading and mathematics), which we also consider in this paper. These interventions are sometimes more effective in a certain domain. Third, we have incorporated potential mechanisms for why these interventions are expected to work. Lastly, we have incorporated an additional table related to all program characteristics, as well as more information about the programs in the methods section. Previously, the latter information could be found in the SI appendix; however, incorporating this into the manuscript might contribute to the discussion on effective interventions more directly.*

**Response to reviewer 1**

*Thank you for your valuable suggestions and feedback on the manuscript, and in particular your insightful comments on the literature section. In response to this comment, we have provided a more detailed description of the interventions, which we believe will improve the overall clarity of the paper. We appreciate your time and effort in reviewing our manuscript and providing constructive feedback.*

- Research Question: Ensure that the research question is explicitly stated.

*The research questions are now more clearly stated in the paper, specifically in the second to last paragraph of the introduction. The research questions we address in this paper are:*

a) *Who participated in the remediation programs? Which students were selected?*
b) *What is the relationship between participation in remediation programs and student achievements?*
c) *Which remediation programs are most effective in improving student achievement?*

- Sample Size: On page 6, correct the sample size to 66,500 (not 66.500).

*Thank you for pointing this out, we have now changed this.*

- Literature Review Citations: In the introductory paragraph of the literature review on page 6, support claims about existing research and numerous studies with proper citations in the main text and reference section.

*Thank you for this suggestion. We have now supported our claims with additional research. Several large-scale review studies, such as Donelly & Patrinos (2022), Hammerstein et al. (2021), and Betthäuser et al. (2022), have shown that students faced large losses after the COVID-19-induced school closures. To provide a comprehensive summary without having to go into detail of every individual study (which for reasons of space is not desirable, given that that is not the focus of our study), we look at review studies that examine many papers regarding initial learning losses after school closures in many countries around the world. The introductory page of the literature review now includes these and more references.*

- Intervention Examples: In the second paragraph of the literature review, on page 6, list specific examples of various interventions. Clarify which interventions, such as one-on-one tutoring, are most effective.

*Thank you for this suggestion. In the literature review section, we now examine various interventions, highlighting one-on-one tutoring as the most effective, supported by average effect sizes. The remainder of this section examines additional interventions, each accompanied by their respective average effect sizes. This allows for a clear comparison of the most and least effective interventions. These discussions can be found from the third paragraph onwards of the literature section.*

*Additionally, in relation to other points raised regarding the literature section, we have included additional studies to enhance the literature review of program characteristics. Furthermore, our answer to your remark 7 provides a detailed description of these enhancements.*

- Areas of Target in Intervention: Explicitly describe the particular areas targeted by the intervention.

*Thank you for this question. We would be happy to do this, however, there is not *one* intervention, but several. Hence, the short answer to this question is that it depends on the intervention. The main target area we are interested in investigating is how these interventions affect students' reading and mathematics performance. In the literature section, we have now mentioned the specific areas of language and mathematics and the expected effect sizes based on previous studies. However, some of the interventions have (additional) different target areas, that we do not focus on in this study.*

*Furthermore, to provide more clarity on the specific remediation programs offered, we added a table to the methodology section to show all the different elements of the programs. In the previous version of our paper, this table with descriptive statistics on the student level provides all the remediation program content could be found in the Supplementary Information (Table S3.4). This table has now been included in the main paper as Table 2. The descriptives in Table 2 show the extra efforts placed on many different remediation programs, such as on behaviour and communication or buying new methods; it also shows which goals the school set for the remediation programs, such as a language goal, mathematics goal, or study-skill goal. It also gives insight into the organisation of these programs, such as the timing, the instructors, or group sizes.*

*For these analyses, we have a total of N = 21,403 students included. Of these students, N = 17,408 students are within schools who have remediation programs but are not participating. These are our control group from the previous analyses. Besides, we have N = 3,959 students who participate in the remediation programs. Of these students, it differs whether they follow a program with or without a certain element. This table shows the descriptive statistics of the participating students (N = 3,959)*

**Table 2.** Descriptive statistics about the elements of remediation programs.

|  | **% of students with element** | **% of students without element** |
| --- | --- | --- |
| New methods | 30.59% | 69.41% |
| Behaviour and communication | 15.91% | 84.09% |
| Automation and guided practice | 6.42% | 93.58% |
| Remedial teaching | 8.28% | 91.72% |
| Professionalization teachers | 2.73% | 97.27% |
| Parental involvement | 0.78% | 99.22% |
| Language goal | 82.29% | 17.71% |
| Math goal | 70.04% | 29.96% |
| Study skills goal | 13.08% | 86.92% |
| Socio-emotional goal | 32.81% | 67.19% |
| Timing of remediation program |  |  |
| *Program outside regular hours* | 13.08% |  |
| *Program during regular hours* | 11.11% |  |
| *Program outside & during* | 27.96% |  |
| *Unknown* | 47.84% |  |
| Executor of remediation program |  |  |
| *Internal executor(s)* | 42.33% |  |
| *External* | 4.65% |  |
| *Internal & external executors* | 50.82% |  |
| *Unknown* | 2.20% |  |
| New or existing program |  |  |
| *New program* | 36.78% |  |
| *Extension of existing program* | 21.22% |  |
| *Unknown* | 40.79% |  |
| Group size |  |  |
| *Individual* | 2.10% |  |
| *Individual and small groups* | 11.42% |  |
| *Small groups* | 6.59% |  |
| *Large groups* | 1.74% |  |
| *Groups of unknown size* | 21.62% |  |
| *Entire classes* | 3.71% |  |
| *Unknown* | 52.82% |  |

- Citations for Previous Research: The second sentence of the last paragraph on page 8 needs citations for statements related to previous research. Include corresponding references in the reference section.

*Thank you. A citation has been added. Research by f.e. Lendrum & Humphrey (2012) shows that implementing interventions is important. By this, they mean that an intervention has a set of specific guidelines that must be followed to implement the intervention optimally. If this is done differently, we cannot expect the same (positive) results.*

- Thematic Review of Learning Loss and Remediation: Enrich the literature review by analyzing learning loss thematically and discussing specific themes related to remediation.

*Thank you for this suggestion. We have written this paper from the starting point that learning loss due to the COVID-19 pandemic is a well-established phenomenon. Several large-scale review studies have shown that students faced large losses after the COVID-19-induced school closures, for example, Donelly & Patrinos (2022), Hammerstein et al. (2021), and Betthäuser et al. (2022). To maintain focus and be mindful of the number of pages of the paper, we have briefly addressed this in the introduction rather than dedicating an extensive section of the literature review to the pandemic and the mechanisms of learning loss. Instead, we have provided a discussion on COVID-19-related learning losses in the initial two paragraphs of the literature review.*

*In response to your feedback, we have revised the literature review on interventions and incorporated additional literature as per remark 4. We agree with the suggestion to enhance the conceptual discussion of various interventions and programs to counter learning loss post-COVID-19 school closures. While we recognise the value of incorporating a theoretical framework, the diverse range of interventions—each with distinct pedagogical and didactical aspects—makes describing an elaborate theoretical framework for each intervention a paper in itself. Hence, we have opted not to include that as such. Instead, we have expanded the current review, offering more comprehensive information on each intervention and detailing their effectiveness in specific areas, such as reading and mathematics. These remediation programs aim to provide students requiring additional support with personalised instruction and tailored education to help them catch up. The expanded literature review explores the methods used to achieve this, including, to name a few, classroom management strategies, computer-assisted programs, and peer-tutoring.*

- Method Section Variables: Enhance the method section by explicitly describing variables such as participation in the remediation program, impact of the program, and effective remediation strategies. Provide a theoretical framework for clarity.

*Thank you for pointing out that this was not clear. An elaborate description of the remediation programs was part of the SI material in the previous version of the paper. The following section has been added to the manuscript: “For roughly 30,000 students, we have information about the school's program. We have the following information regarding the remediation programs: whether the remediation program purchased new methods or techniques for teaching and learning; whether the remediation program focused on behaviour and communication; whether the remediation program focused on automation and guided practice; whether the remediation program offered remedial teaching; whether the remediation program had a language goal, mathematics goal, study-skills goal, or socio-emotional goal; the timing of the program taking place outside of during normal hours; whether the program was executed by internal, external or a combination of internal and external staff; the group size of the remediation program; whether the remediation program originates from a new program or an established program.”*

*Additionally, we have, in relation to remark 5, provided the manuscript with an additional table (Table 2 in the manuscript) concerning the content of the remediation programs. This further shows the different remediation strategies and content of the programs.*

- Data Analysis Tools: Specify the tools used for data analysis in the method section of the main text.

*If we understand this question correctly, do you want us to add which statistical programme we used and which commands? We have now added that to the sub-section “Analyses”. We estimate equation 1 with the logistic package and equations 2 and 3 with the xtreg package in Stata 15.*

- Policy and Pedagogical Implications: Conclude the discussion section by adding policy and pedagogical implications based on the study’s findings.

*Thank you for your feedback. In response to your suggestion to conclude the discussion section by adding policy and educational implications based on the study's findings, we include the following considerations: the importance of supporting and funding targeted remediation efforts and recognising the role of school autonomy in addressing educational inequalities. These two considerations have been added to the conclusion and discussion.*

*“Our study shows that remediation programs that address content gaps due to the COVID-19 pandemic can be effective. This approach is not new; however, the rapid implementation of these programs and the diversity of schools have resulted in varying levels of success. Although not universally effective, many programs have shown enough promise to justify allocating funds for their development. Furthermore, the results show that schools can manage this autonomy in the Dutch context, where schools enjoy considerable autonomy. Our study shows that the students who most needed support were correctly identified and enrolled in these remediation programs. Furthermore, schools were able to develop and implement programs tailored to the needs of these students. This suggests a robust capacity within schools to use their autonomy in ways that benefit students, particularly in challenging circumstances such as those posed by the pandemic.”*

*Thank you once again for your valuable feedback. We hope we have been able to deal with your comments satisfactorily.*

**Response to reviewer 2**

*Thanks for your thoughts and feedback on our paper. Especially with respect to major point 2, we agree that the analyses and interpretation of the research aims could be better articulated. Your comment has caused us to rethink our strategy for research goal 3 and analyse it in a different, improved, way.*

**Major point 1.**

Analyses and interpretation of research aim (2): To estimate the overall effect of the national remedial education program on children’s test performance in math and reading, the manuscript compares the performance gains of students who participated in the program and those who did not, between June 2020 and June 2021. Given that students who participated in the program had a lower baseline performance and were from more disadvantaged backgrounds, estimating overall effects in this difference-in-difference design requires that the learning rate of the two comparison groups is similar prior to the program. The manuscript acknowledges the importance of this parallel trends assumption and refers to section 7 of the Supplementary Information where this assumption is tested. While I agree that figures S7.1-4 suggest that learning progress is similar across the two comparison groups for most time periods, there is a notable divergence in the trends in reading progress between January/February 2020 and June 2020 for cohorts 1 and 2 (see F7.1-2 on p42-43 of the SI). Arguably, this time period is most relevant, as it is right before the remedial education program was implemented. This divergence in learning progress is not surprising, as it reflects early learning losses during the COVID-19 pandemic in the Netherlands (see Haelermans et al. 2022 and Engzell et al. 2021). These learning losses are known to be concentrated amongst the groups of students from more disadvantaged backgrounds, who also had a higher likelihood of participating in remedial education programs, as shown by the manuscript in the analyses for research aim (1). It would be important to take this divergence into account when interpreting the results for research aim (2). The divergence in learning progress may suggest that the estimates of the overall effects of the remedial education program are lower-bound estimates, as it is likely that the learning rate of children with a lower baseline performance and from lower socioeconomic backgrounds would have continued to have a(n even) lower learning rate in the absence of the remedial education program.

*Thank you for the suggestion to incorporate this in the manuscript. We have added it in the results section, where the pre-trends are first discussed, and we added it to the conclusion and discussion section.*

*At the start of the results section, we discuss the pre-trends. In the paragraph where this is discussed, we added the following sentence: “In the pre-trends, there is some divergence in progress between students who participated in the programs and non-participating students, reflecting the obvious differences in early learning losses after the first school closures, making it important to consider that the overall effects of the program might be lower-bound estimates.”*

*In the conclusion and the discussion section, we list several points as to why the effect sizes we find are considerable, given the situation. In this section, we highlight your suggestion as well. We added the following paragraph: “Fifth, given the divergence in the pre-trends regarding test score progress between January/February 2020 and June 2020 among participating and non-participating students, it is important to consider that the overall effects of the program might be lower-bound estimates. This divergence reflects the early learning losses during the COVID-19 pandemic, suggesting that participating students (lower-performing students from disadvantaged backgrounds) would likely have continued to fall further behind without the remediation programs.”*

**Major point 2:**

Analyses and interpretation of research aim (3): As its third main aim, the manuscript seeks to examine how program effects co-vary with the characteristics of specific remedial education measures implemented in different schools. To this end, authors run a series of 13 models in which they successively replace the binary treatment/control dummy variable used for the analyses of research aim (2) with multi-category measures of different characteristics across which different remedial education measures vary and which are thought to be relevant for children’s learning progress. The results from these models are shown in Tables S6.1-13 in the SI. They then select three of these characteristics (remedial teaching, group size, and staff) and combine them in the coefficient plot shown in Figure 2.

It is not clear to me that the chosen analytical approach is best suited to achieve the third research aim of the study and I suggest an alternative approach further below. A key problem is that the characteristics of the remedial education measures implemented in different schools are likely to co-vary and be confounded by characteristics that are not included in each of the separate models shown in Tables S6.1-13. This may explain counterintuitive results such that remedial education measures with a focus on language have no positive effect on children’s reading progress, but do have an effect on children’s math progress (see Table S6.8 on p.35 of the SI). In short, not including different program characteristics in the same model considerably limits the extent to which the associations shown between effect sizes and program characteristics can be interpreted as causal relationships. It would be important to highlight this in the text.

A related problem of the current analytical approach is that one cannot gauge the statistical significance of the differences in the effect sizes between different remedial education measures. This is because students who participated in a given remedial education measure are compared to students who did not participate in any remedial education program, rather than students who participated in a remedial education measure that differed on the relevant characteristic in question. Relatedly, the current interpretation of some of results of Figure 2 reported on p.15-16 are not technically correct. While the interpretation concerning ‘remedial teaching’ is correct, the interpretation of the results concerning group size should be adjusted. Currently the manuscript states that “… positive outcomes were associated with small-group programs as opposed to larger settings, with a 0.26 SD higher increase in the test scores for participants with small-group remediation programs.” This is somewhat misleading, as it suggests that there is a substantively large and statistically significant difference in the effects of remedial education measures characterised by small groups, vs larger groups. Yet, as Figure 2 shows, the difference between ‘entire class’ and ‘small groups’ is substantively relatively small and (telling from the confidence intervals) not statistically significant. It would be important and relatively straightforward to adjust the interpretation to reflect the fact that the reference group here are students who did not participate in remedial education and there seems to be no statistically significant difference in the effectiveness of remedial education measures with different group sizes.

An alternative analytical approach to address research aim (3) would be to extract effect sizes of the remedial education program for each school in the sample and use these as the dependent variable with program characteristics as the focal independent variables, controlling for school characteristics and characteristics of the student body participating in the remedial education program. Effect sizes could be weighed according to the precision of each estimate to account for cross-school variation herein. An important advantage of this alternative analytical approach would be that one could include all relevant program characteristics in the same model and thereby avoid the confounding between program characteristics that likely limits the interpretability of the results in the current version of the manuscript. The proposed alternative approach would also show the substantive size and statistical significance of the association between different program characteristics and children’s learning progress. It would also facilitate jointly showing and discussing the coefficients of all program characteristics analysed rather than focussing on only three characteristics in the main text, as is currently done.

It would be helpful to explain more clearly what is meant by the concepts used to describe the different program characteristics (e.g. what is meant by ‘remedial teaching’), as well as how they are operationalised and measured. Relatedly, it would be helpful to add more theoretical discussion of why the characteristics of different remedial education measures that are examined are thought to be relevant.

*Thank you for your feedback on the analyses concerning the third research objective and the suggested alternative approach. We acknowledge that remediation programs' contents are interrelated and that several remediation characteristics may co-vary. Therefore we first followed the suggested approach by extracting average student scores at the school level for the 2020/2021 academic year when the remediation programs were implemented. This was possible for around 65 schools for which we had detailed information on the characteristics of the remediation programs. The number of schools (N = 65) can be explained by the fact that, we were able to carry out most of the analyses of the remediation programs on 125-150 clusters (school * year). A given school in year 2019/2020 is a cluster, while the same school in year 2020/2021 is a cluster as well. This provides information for 65 schools when all remediation program characteristics are included in a single model. For these 65 schools we analyse the content of the remediation programs with the extracted test scores for composite, reading and mathematics. Table R1 below shows the results at the school level.*

*However, the challenges we encountered with this approach suggest that it may not be the most effective way of dealing with the confounding nature of program characteristics. The first issue is the sample size: Conducting analyses at the school level with only 65 schools led to power problems as the alternative approach yielded a very small sample. More importantly, remediation programs were offered to only 10-20% of students, meaning that school-level effect sizes do not accurately reflect the efforts of this small group of participants.*

*The results of Table R1 show very little to no significant effects of the content of remediation programs on either the composite score, reading score or mathematics score. We find one positive relationship between internal staff and test scores. All additional variables show no significant effects. Besides, the relationship between a mathematics goal and mathematics test scores show a negative, not significant effect, which is a counterintuitive relationship.*

*However, as we recognized the importance of considering these factors, we have hence decided to use a different strategy (both different to your suggested method as well as to the method we applied in the previous version of the paper). We have chosen to include these remediation program characteristics in our student-level difference-in-differences analysis. Our revised approach involves including all remediation program characteristics as control variables. This strategy allows us to keep the analyses at the student level while at the same time keeping statistical power when including all variables in the model. Furthermore, this allows us to differentiate between participating and non-participating students. Some of the main conclusions are that we find positive effects for small groups in the remediation programs of 2-5 or 6-10 students and positive effects for mathematics goals on mathematics test scores. Please see the revised tables in the appendix of the paper, where all remediation program characteristics have been analysed with all the control variables.*

*We hope this approach aligns with the comments you raised in proposing the alternative approach. We are confident this is a more suitable approach to take into account the co-varying aspects of the different remediation programs.*

***Table R1. Alternative approach to study the content of the remediation programs in relation to the student’s test scores.***

|  | ***Composite score*** | ***Reading score*** | ***Mathematics score*** |
| --- | --- | --- | --- |
| Extra remedial teaching | 0.029 | 0.006 | 0.051 |
|  | (0.126) | (0.132) | (0.122) |
| Small groups | 0.114 | 0.132 | 0.095 |
|  | (0.193) | (0.202) | (0.187) |
| Large groups | 0.164 | 0.196 | 0.132 |
|  | ( 0.184) | (0.193) | (0.178) |
| Individual | 0.102 | 0.083 | 0.120 |
|  | (0.233) | (0.245) | (0.226) |
| Individual and in groups | 0.136 | 0.148 | 0.125 |
|  | (0.181) | (0.190) | (0.176) |
| External staff | 0.089 | 0.093 | 0.085 |
|  | (0.224) | (0.235 | (0.217) |
| Internal staff | 0.181* | 0.190* | 0.171* |
|  | (0.080) | (0.084) | (0.078) |
| Unknown staff | 0.478 | 0.535 | 0.421 |
|  | (0.326) | (0.342) | (0.316) |
| Additional methods | 0.015 | -0.010 | 0.041 |
|  | (0.102) | (0.107) | (0.099) |
| Focus on behavior and communication | 0.267 | 0.294 | 0.240 |
|  | (0.187) | (0.197) | (0.182) |
| Extra automation and guided practice | 0.067 | 0.056 | 0.078 |
|  | (0.144) | (0.151) | (0.139) |
| Goal language | 0.045 | 0.030 | 0.060 |
|  | (0.124) | (0.130) | (0.120) |
| Goal mathematics | -0.132 | -0.133 | -0.131 |
|  | (0.097) | (0.102) | (0.094) |
| Goal study skills | -0.155 | -0.156 | -0.154 |
|  | (0.124) | (0.130) | (0.121) |
| Goal socio-emotional | -0.007 | -0.023 | 0.009 |
|  | (0.106) | (0.111) | (0.102) |
| Unknown timing of program | -0.192 | -0.172 | -0.212 |
|  | (0.183) | (0.192) | (0.177) |
| During and outside regular education hours | -0.169 | -0.156 | -0.182 |
|  | (0.179) | (0.188) | (0.174) |
| During regular education hours | -0.015 | 0.004 | -0.034 |
|  | (0.201) | (0.211) | (0.195) |
| Unknown program content | 0.007 | 0.015 | -0.001 |
|  | (0.088) | (0.093) | (0.086) |
| Extension of existing program | -0.067 | -0.073 | -0.061 |
|  | (0.119) | (0.125) | (0.115) |
| Constant | -0.155 | -0.188 | -0.122 |
|  | (0.259) | (0.272) | (0.251) |
| Observations | 65 | 65 | 65 |
| R-squared | 0.332 | 0.324 | 0.337 |

**Minor points:**

- Minor point 1: The confidence intervals shown in Figure 1 and Figure 2 seem to reflect 0.10 thresholds for statistical significance rather than the usual 0.05 thresholds (see, e.g., Tale S6.1). Given that this is relatively unconventional, it would be important to highlight this in the main text and in the description of the figures.

*Thank you for pointing this out. Figure 1 reflects at the 5% significant threshold; however, indeed, Figure 2 reflected the 10% significance level. As we have altered the analyses related to Figs 1a, 1b and 2, the statistical significance thresholds of Figure 2 have now been altered as well to the 5% significance levels.*

- Minor point 2: It would be helpful to show results in Figure 2 for math and reading separately, given that effects vary by subject domain (as shown in Tables S6.1-13).

*Thank you for highlighting the difference between reading and mathematics. Indeed, in the old version of the manuscript the effect sizes for the remediation programs were only reported for the composite score, while the separate analyses for reading and mathematics were only shown in the supplementary information.*

*In the new version, we have made the distinction between average effects (composite score), and the individual effects for mathematics and reading. Below is an example of a figure for “group size”.*

- Minor point 3: It is not clear why the model with school-level fixed effects shown in Table S5.5 is not used as the preferred model in the main text for research aim (2). This would seem to be the most robust specification.

*Thank you for this question. We have now made this the preferred specification; in hindsight, we agree that it is indeed the most robust specification. Table 3 in the main text now includes the FE models, and the SI Tables S5.1, 5.2, and 5.3 show the elaborate models for the composite score, reading score, and mathematics score separately, all with FE included. The original Table S5.5 has been deleted.*

**Table 3: Difference-in-Differences analysis for participants and non-participants.**

|  | **Composite** | **Reading** | **Mathematics** |
| --- | --- | --- | --- |
| School year 2020/2021^a^ | 0.010 | 0.015^ | 0.006 |
|  | (0.007) | (0.008) | (0.008) |
| Participating students^b^ | -0.537*** | -0.514*** | -0.560*** |
|  | (0.014) | (0.016) | (0.016) |
| School year * Participation | 0.052** | 0.050** | 0.054** |
|  | (0.017) | (0.019) | (0.019) |
|  |  |  |  |
| Student controls | Yes | Yes | Yes |
| School level controls | Yes | Yes | Yes |
| School-level fixed effects | Yes | Yes | Yes |
|  |  |  |  |
| Constant | -0.242* | -0.447** | -0.036 |
|  | (0.123) | (0.140) | (0.140) |
|  |  |  |  |
| Observations | 66,439 | 66,439 | 66,439 |
| R-squared | 0.138 | 0.121 | 0.140 |
| Clusters | 456 | 456 | 456 |
| Note: Robust standard errors in parentheses; *** p < 0.001, ** p < 0.01, * p < 0.05, ^ p < 0.1. ^a^ the reference category is the school year 2019/2020; ^b^ the reference category is students who did not participate in the remediation programs but are enrolled in schools that offer remediation programs. Student controls include sex, migration background, parental education and income, and household structure, and school-level controls include denomination, urbanization, and the disadvantage score of the school. | | | |

- Minor point 4: The notes under Figure 1 state that “Students’ achievement is measured by the combined score of reading and mathematics on the difference between the midterm test and the end-of-year test in the school year 2019/2020.” This is not very clear and it would be helpful if it could be made clearer in the figure legend and main text what is meant here.

*Thank you for your comment. The notes under the Figure have been adapted as well as the text explaining the results. In brief, schools received funding for 10% (or 20%) of their students to participate in remediation programs. In particular, students with the greatest learning losses at the time of the COVID-19 school closures were targeted. We included a measure of 'learning loss' as an explanatory variable in the model. This "learning loss" is measured by subtracting the score at the end of the 2019/2020 school year from the score at mid-year 2019/2020 (just before the pandemic started). This score is calculated separately for reading and mathematics and later combined into a composite score. We have included this variable in the model under 'prior achievement'. In the SI file, we repeated the analyses with a measure of learning loss separately for reading and mathematics (see Table S4.1). We also analysed participation in the remediation programs using only the score at the end of the 2019/2020 school year (see Table S4.2).*

- Minor point 5: It would be interesting and aid the interpretability of results to show descriptive statistics on the prominence of different characteristics of the remedial education measures chosen by different schools in the Netherlands. For instance, how many schools employed small group measures as opposed to measures with larger groups of students?

*Thank you for your comment. To clarify the specific remediation programs offered, the table below (Table 2) is added to the methodology section in which we show the different elements of the programs. This table for example shows the % of students who followed a remediation program that placed extra efforts on behaviour and communication or buying new methods; it also shows which goals the school set for the remediation programs, such as a language goal, mathematics goal, or study-skill goal. It also gives insight into the organisation of these programs, such as the timing, the instructors, or group sizes in which the program is structured.*

*Unfortunately, providing this information at the school level is impossible, as we are not allowed to report at the school level when N < 10 in subgroups. Some programs, or the content of the remediation programs, are offered at fewer schools. Furthermore, it is also more in line with our other analyses to report the different characteristics at the student level, as all our other analyses are at the student level.*

**Table 2.** Descriptive statistics about the elements of remediation programs.

|  | **% of students with element** | **% of students without element** |
| --- | --- | --- |
| New methods | 30.59% | 69.41% |
| Behaviour and communication | 15.91% | 84.09% |
| Automation and guided practice | 6.42% | 93.58% |
| Remedial teaching | 8.28% | 91.72% |
| Professionalization teachers | 2.73% | 97.27% |
| Parental involvement | 0.78% | 99.22% |
| Language goal | 82.29% | 17.71% |
| Math goal | 70.04% | 29.96% |
| Study skills goal | 13.08% | 86.92% |
| Socio-emotional goal | 32.81% | 67.19% |
| Timing of remediation program |  |  |
| *Program outside regular hours* | 13.08% |  |
| *Program during regular hours* | 11.11% |  |
| *Program outside & during* | 27.96% |  |
| *Unknown* | 47.84% |  |
| Executor of remediation program |  |  |
| *Internal executor(s)* | 42.33% |  |
| *External* | 4.65% |  |
| *Internal & external executors* | 50.82% |  |
| *Unknown* | 2.20% |  |
| New or existing program |  |  |
| *New program* | 36.78% |  |
| *Extension of existing program* | 21.22% |  |
| *Unknown* | 40.79% |  |
| Group size |  |  |
| *Individual* | 2.10% |  |
| *Individual and small groups* | 11.42% |  |
| *Small groups* | 6.59% |  |
| *Large groups* | 1.74% |  |
| *Groups of unknown size* | 21.62% |  |
| *Entire classes* | 3.71% |  |
| *Unknown* | 52.82% |  |

- Minor point 6: It would be helpful to note that the analyses in Table S5.6 suggest that there are no differences in the effectiveness of the remedial education program across age groups. This could also be tested directly, using the alternative analytical approach outlined above.

*Thank you for your comment. The analyses per grade have been updated including the Fixed Effects (FE) analyses as the preferred model specification. The old Table S5.6 has been updated. The results of the FE analyses suggest that higher grades show higher and significant increases, while for the youngest age group, no significant effects were found. We have added the Table below for clarity. As the robustness check with FE has become the main analysis, Table S5.6 can now be found in the SI as Table S5.4*

**Table S5.4**. DiD-estimator for analyses separately per grade on students’ achievements before and after remediation programs.

|  | **Composite** | **Reading** | **Mathematics** |
| --- | --- | --- | --- |
| 2^nd^ grade to 3^rd^ grade | 0.038 | 0.043 | 0.037 |
|  | (0.029) | (0.033) | (0.033) |
| 3^rd^ grade to 4^th^ grade | 0.065** | 0.075** | 0.054^ |
|  | (0.023) | (0.026) | (0.029) |
| 4^th^ grade to 5^th^ grade | 0.065* | 0.061* | 0.070* |
|  | (0.027) | (0.031) | (0.030) |
| Robust standard errors in parentheses; *** p < 0.001, ** p < 0.01, * p < 0.05, ^ p < 0.1. ^a^ the reference category is the school year 2019/2020; ^b^ the reference category is students who did not participate in the remediation programs but are enrolled in schools that offer remediation programs. Student controls include sex, migration background, parental education and income, and household structure, and school-level controls include denomination, urbanization, and the disadvantage score of the school. | | | |

- Minor point 7: It would be helpful to add relevant references to the discussion of the existing evidence on remedial education programs on p.6-8. For instance what evidence does the reported effect size of 0.4 SD for one-to-one tutoring on p.7 (top) refer to?

*Thank you for your comment. Translating the 0.4 SD to a more tangible way of what exactly this means is a good addition to the paper. In the literature framework, we now have added translations of effect sizes in standard deviations, to effect sizes expressed in additional weeks of learning. For example, the following information has been added to the manuscript: “The average effect size is estimated to be around 0.49 SD increase in attainment or roughly six months of additional progress. On average, one-to-one tutoring seems more effective for reading (+0.49 SD) than mathematics (+0.14 SD).” Besides, according to the Kraft (2020), effect sizes below 0.05 SD are considered small, effect sizes between 0.05 SD and 0.20 SD are considered moderate, and above 0.20 SD are considered large. This reference has been added to the discussion. Nevertheless, the reported effect sizes in the literature review are mostly from studies conducted before the COVID-19 pandemic in a more controlled study. We expect lower effect sizes based on our study.*

- Minor point 8: It would be helpful to state more clearly the three main aims/research questions in the introduction of the manuscript.

*The research questions are now more clearly stated in the paper, specifically in the second to last paragraph of the introduction. The research questions we address in this paper are:*

- *Who participated in the remediation programs? Which students were selected?*
- *What is the relationship between participation in remediation programs and student achievements?*
- *Which remediation programs are most effective in improving student achievement?*
- Minor point 9: Using predicted probabilities rather than odds ratios may aide the interpretability of Figure 1.

*Thank you for this suggestion. In the previous version, Figure 1 showed the odds ratios from the logistic regression. This showed that students with certain characteristics have a higher or lower chance of being selected for remediation programs.*

*The suggested option of showing predicted probabilities will slightly differ in results and interpretability. With this option, we show the average chance of being selected based on the other characteristics, as the predicted probabilities consider the relation with the other variables.*

*We recognize that predicted probabilities might offer advantages in interpretation, especially related to the test scores. Below and in the paper, these figures can be found have included the figure with predicted probabilities in the response to the reviewers.*

*Please see figs 1a and 1b below.*

Likelihood to participate - student background characteristics

|  |  |
| --- | --- |
| Boy vs mean | -0.0031263 |
| Girl vs mean | 0.0031263 |
| Grade 2 vs mean | 0.0055561 |
| Grade 3 vs mean | -0.0086116 |
| Grade 4 vs mean | 0.0030554 |
| Dutch vs mean | -0.0040624 |
| Non-western background vs mean | 0.0036874 |
| Western background vs mean | 0.000375 |
| Low educated vs mean | 0.0158284 |
| Average vs mean | -0.0019045 |
| High vs mean | -0.0139239 |
| Low income vs mean | 0.005552 |
| Average income vs mean | -0.0016952 |
| High income vs mean | -0.0038568 |
| Both parents work vs mean | -0.0094026 |
| Only father works vs mean | -0.0045252 |
| Only mother works vs mean | 0.0058731 |
| Both parents do not work vs mean | 0.0080547 |
| Two-parent household vs mean | 0.0021035 |
| One-parent household vs mean | -0.0021035 |

***Fig 1a: Likelihood of participating in remediation programs based on student background characteristics.***

Likelihood to participate - student test scores

|  |  |
| --- | --- |
| -6 SD | 0.915695 |
| -5 SD | 0.843739 |
| -4 SD | 0.728711 |
| -3 SD | 0.572142 |
| -2 SD | 0.399744 |
| -1 SD | 0.249006 |
| 0 SD | 0.141606 |
| 1 SD | 0.0758001 |
| 2 SD | 0.0391589 |
| 3 SD | 0.019843 |
| 4 SD | 0.0099546 |

***Fig 1b: Likelihood of participating in remediation programs based on previous performance.***

*Thank you once again for your valuable feedback. We hope we have been able to deal with your comments satisfactorily.*

***References***

*Betthäuser, B., Bach-Mortensen, A., & Engzell, P. (2022). A systematic review and meta-analysis of the impact of the COVID-19 pandemic on learning. LIEPP Working Paper, (134).*

*Donnelly, R., & Patrinos, H. A. (2022). Learning loss during Covid-19: An early systematic review. Prospects, 51(4), 601-609.*

*Haelermans, C., Huijgen, T., Jacobs, M., Levels, M., van der Velden, R., van Vugt, L., & van Wetten, S. (2020). Using data to advance educational research, policy, and practice: Design, content, and research potential of the Netherlands Cohort Study on Education. European Sociological Review, 36(4), 643-662.*

*Hammerstein, S., König, C., Dreisörner, T., & Frey, A. (2021). Effects of COVID-19-related school closures on student achievement-a systematic review. Frontiers in psychology, 12, 746289.*

*Lendrum, A., & Humphrey, N. (2012). The importance of studying the implementation of interventions in school settings. Oxford Review of Education, 38(5), 635-652.*
